# Supplementary material for: Multi-compartmental MOF microreactors derived from Pickering double emulsions for chemo-enzymatic cascade catalysis
Source: Nat Commun. 2023 Jun 3;14:3226. doi: 10.1038/s41467-023-38949-w (PMC10239487; doi:10.1038/s41467-023-38949-w)
Supplement: Supplementary file 1 — Supplementary Information [file 41467_2023_38949_MOESM1_ESM.pdf]

Supplementary Information for

Multi-compartmental MOF microreactors derived from Pickering double emulsions for chemo-enzymatic cascade catalysis

Danping Tian[1], Ruipeng Hao[1], Xiaoming Zhang[1]*, Hu Shi[1], Yuwei Wang[1], Linfeng Liang[2], Haichao Liu[3]* and Hengquan Yang[1]*

[1]*School of Chemistry and Chemical Engineering, Shanxi University, Taiyuan 030006, China*

[2] *Institute of Crystalline Materials, Shanxi University, Taiyuan 030006, China*

[3] *Beijing National Laboratory for Molecular Sciences, College of Chemistry and Molecular Engineering, Peking University, Beijing 100871, China*

*To whom correspondence should be addressed: xmzhang4400@sxu.edu.cn; hcliu@pku.edu.cn; hqyang@sxu.edu.cn

Contents

Supplementary Figure 1. Characterization of the silica emulsifiers with different wettability
Supplementary Figure 2. Characterization of the primary oil-in-water Pickering emulsion
Supplementary Figure 3. Optical micrograph of oil-in-water-in-oil Pickering double emulsions and its droplet size distribution
Supplementary Figure 4. Confocal laser scanning microscopy images of the Pickering double emulsion droplets
Supplementary Figure 5. Standing tests of the Pickering double emulsion
Supplementary Figure 6. Fluorescence microscopy observation of the Pickering double emulsion with outer oil phase dyed by Nile Red
Supplementary Figure 7. Fluorescence microscopy observation of the Pickering double emulsion as a function of time with inner water phase dyed by FITC-Dextran
Supplementary Figure 8. Characterization of the multi-compartmental MOF-74 microreactors
Supplementary Figure 9. SEM images of the multi-compartmental MOF-74 microreactors as a function of metal ion dosage
Supplementary Figure 10. FT-IR spectra of the multi-compartmental MOF-74 microreactor and uncoordinated H_4DOBDC ligand
Supplementary Figure 11. Optical micrographs of the primary oil-in-water and final oil-in-water-in-oil double emulsions prepared with different hydrophilic emulsifier dosages
Supplementary Figure 12. SEM images of the multi-compartmental MOF-74 microreactors prepared with different inner droplet volume fractions
Supplementary Figure 13. SEM images of the multi-compartmental MOF-74 microreactors prepared with different inner droplet volume fractions by an additional pre-coordination process
Supplementary Figure 14. Optical microscopy observations for the formation of multi-compartmental MOF-74 microreactor at different growth times
Supplementary Figure 15. Characterization of the single compartment MOF-74 that derived from water-in-oil Pickering emulsions
Supplementary Figure 16. SEM images of the multi-compartmental MOF-74 microreactors as a function of pre-coordination time
Supplementary Figure 17. Observations of the interior compartments of MOF-74 microreactors that prepared with a pre-coordination process
Supplementary Figure 18. Elemental mappings of various multi-compartmental MOF microreactors
Supplementary Figure 19. FT-IR spectra of various multi-compartmental MOF microreactors

Supplementary Figure 20. N_2 sorption characterization of various multi-compartmental MOF microreactors
Supplementary Figure 21. Large-scale production of multi-compartmental MOF-74 microreactors
Supplementary Figure 22. Molecular structure and size of the probe molecules
Supplementary Figure 23. Confocal fluorescence microscopes and their corresponding intensity profiles of the multi-compartmental MOF-74 microreactors loaded with Rhodamine B-labelled enzymes
Supplementary Figure 24. Stability tests of the enzymatic Pickering double emulsion in polar solvent
Supplementary Figure 25. Characterization of the MOF-74 material synthesized by traditional solvothermal method
Supplementary Figure 26. Confocal fluorescence microscopy of the single compartment MOF-74 microreactor encapsulated with different enzymes
Supplementary Figure 27. SEM images of the multi-compartmental MOF-74 microreactor after loading with Grubbs catalyst and CALB
Supplementary Figure 28. FT-IR spectra of Grubbs catalyst, the multi-compartmental MOF-74 and Grubbs/CALB@MOF-74 solid catalyst
Supplementary Figure 29. Kinetic plots for the two catalytic steps over separated solid catalysts
Supplementary Figure 30. Kinetic plots for ring-closing metathesis of 1,6-heptadien-4-ol over the homogenous mixture of Grubbs/CALB and pure Grubbs' catalyst
Supplementary Figure 31. Catalytic results of Grubbs/CALB@MOF microreactors for the ring-closing metathesis/transesterification cascade reaction at different temperatures
Supplementary Figure 32. Catalytic performance of Grubbs/CALB@MOF in the one-pot cascade reaction of different substrates
Supplementary Figure 33. The stability and recyclability test of Grubbs/CALB@MOF catalyst
Supplementary Figure 34. Chemo-enzymatic cascade catalysis in glucose oxidase (GOx)/Fe-porphyrin driven oxidation reaction
Supplementary Figure 35. Molecular structure and size of ABTS
Supplementary Figure 36. FT-IR spectra of and GOx/TPP(Fe)@MOF, GOx and TPP(Fe)
Supplementary Table 1. Metal salts and organic ligands of the synthesized multi-compartmental MOF microreactors and their corresponding textural properties
Supplementary Table 2. Encapsulation efficiencies of various enzymes and molecular catalysts in different systems
Supplementary Methods

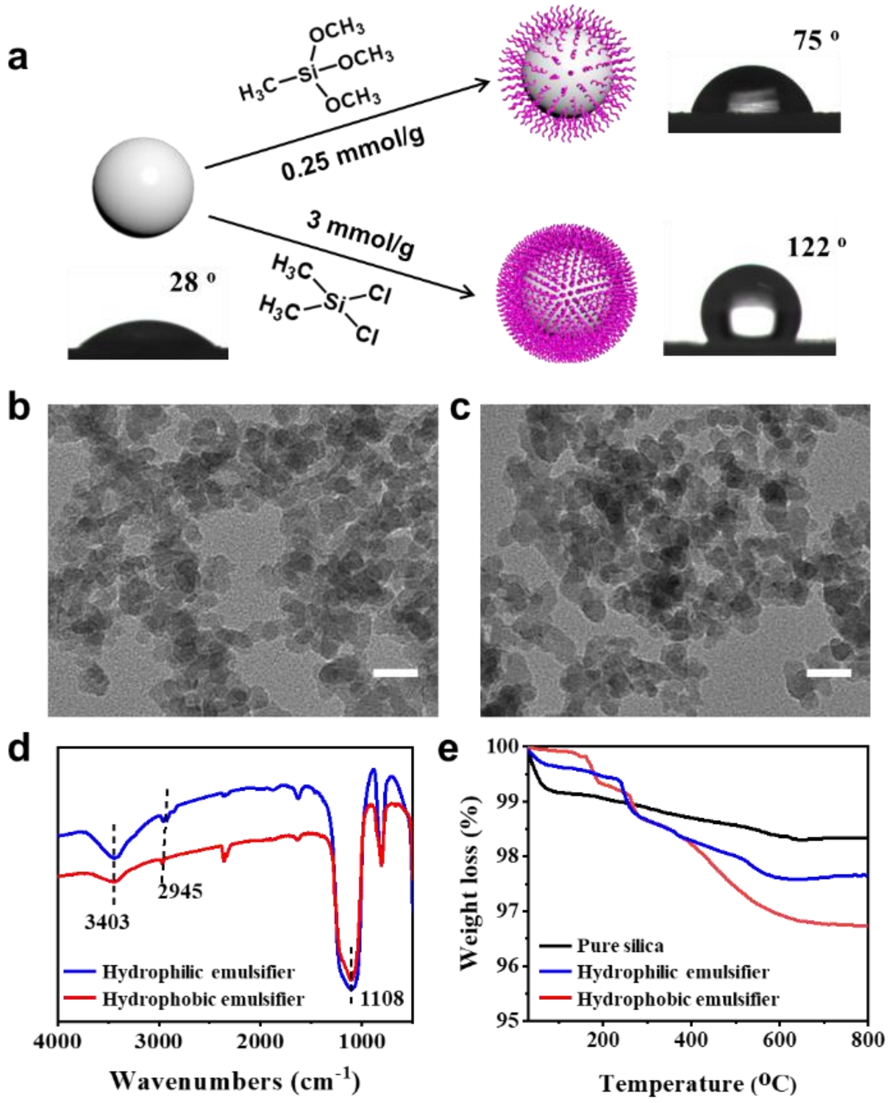

Supplementary Figure 1. Characterization of the silica emulsifiers with different wettability. a, Schematic illustration of the synthesis process based on a commercial silica nanoparticle and the corresponding water contact angles. (**b, c**) TEM images of the hydrophilic and hydrophobic silica emulsifiers, scale bar = 50 nm. **d,** FT-IR spectrum. **e,** TGA curves.

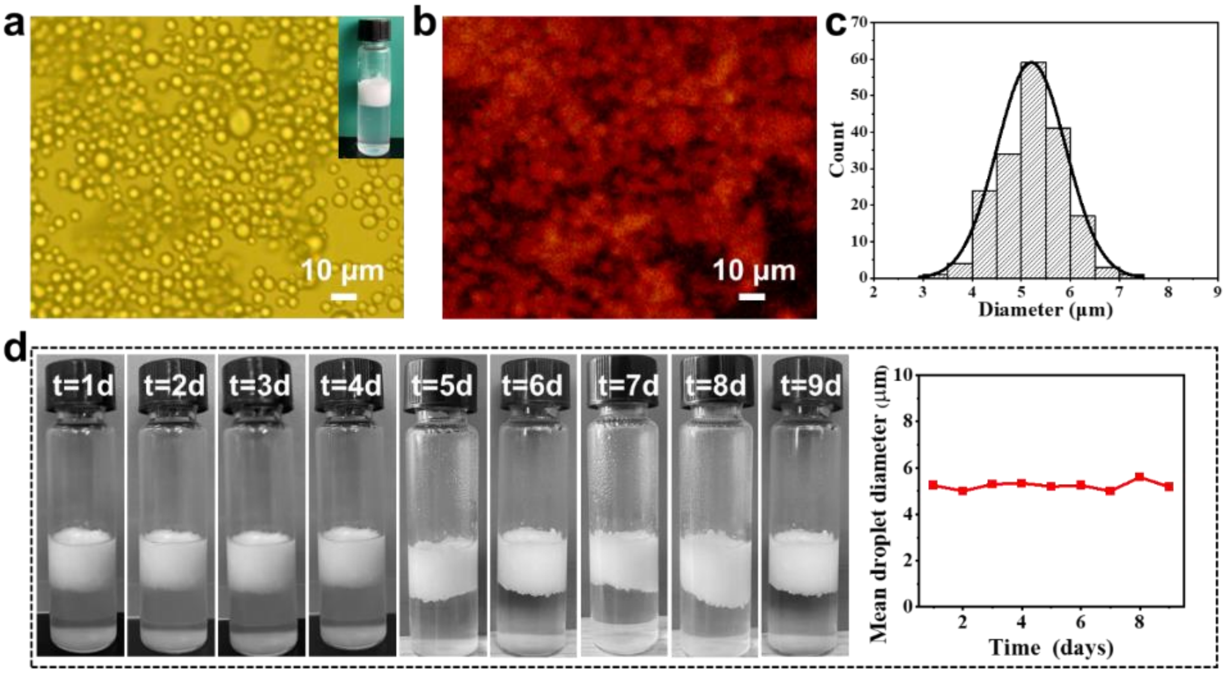

Supplementary Figure 2. Characterization of the primary oil-in-water Pickering emulsion. a, Optical microscopy image of the emulsion droplets (5 wt% solid emulsifier dosage with respect to the inner oil phase). **b**, Fluorescence microscopy observation with inner oil labeled by Nile Red. **c**, The corresponding droplet size distribution. **d**, Stability of the emulsion along with storage time, and their statistic droplet size variation.

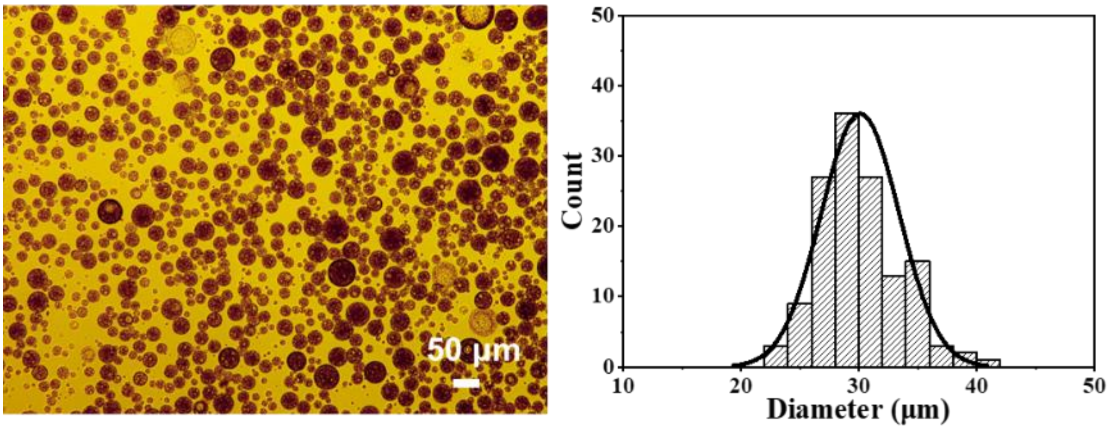

Supplementary Figure 3. Optical micrograph of the oil-in-water-in-oil Pickering double emulsion and its droplet size distribution. Scale bar = 50 μm.

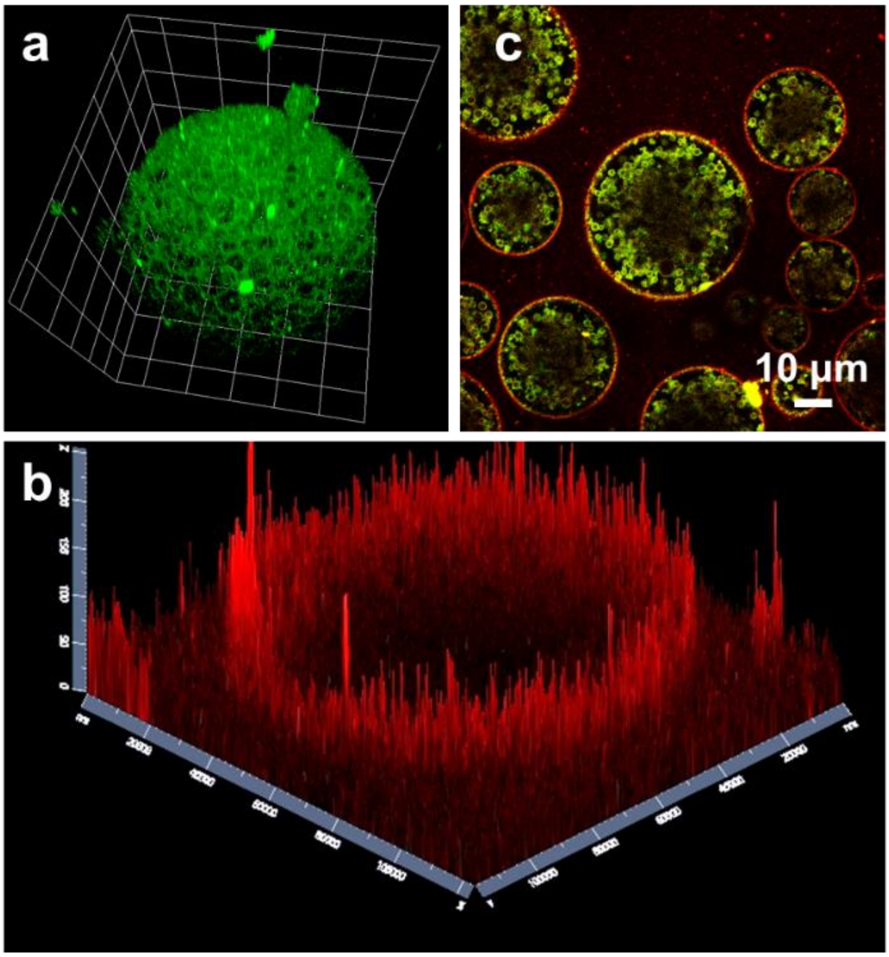

Supplementary Figure 4. Confocal laser scanning microscopy images of the Pickering double emulsion droplets. (**a**, **b**) 2.5D CLSM images of a single droplet with hydrophilic emulsifier labeled by FITC-I or hydrophobic emulsifier labeled by Rhodamine B. **c**, Merged confocal microscopy observation.

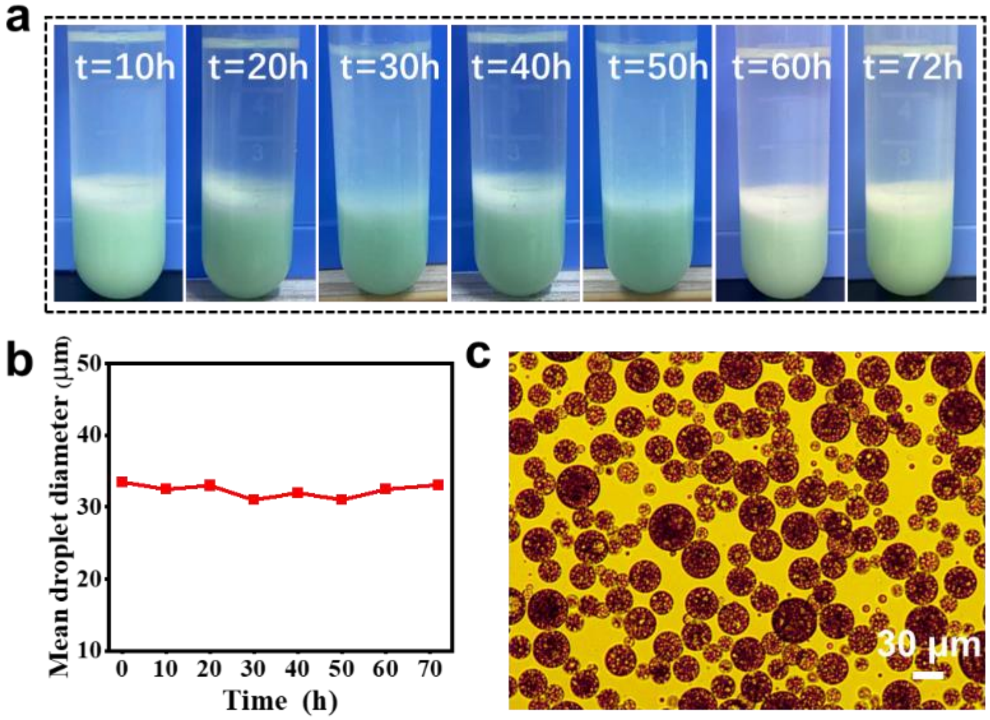

Supplementary Figure 5. Stability tests of the Pickering double emulsion. a, Digital images of the oil-in-water-in-oil emulsions along with storage time. **b**, The statistic droplet size variation as a function of time. **c**, Optical micrograph of the double emulsion droplets after standing for 72 h.

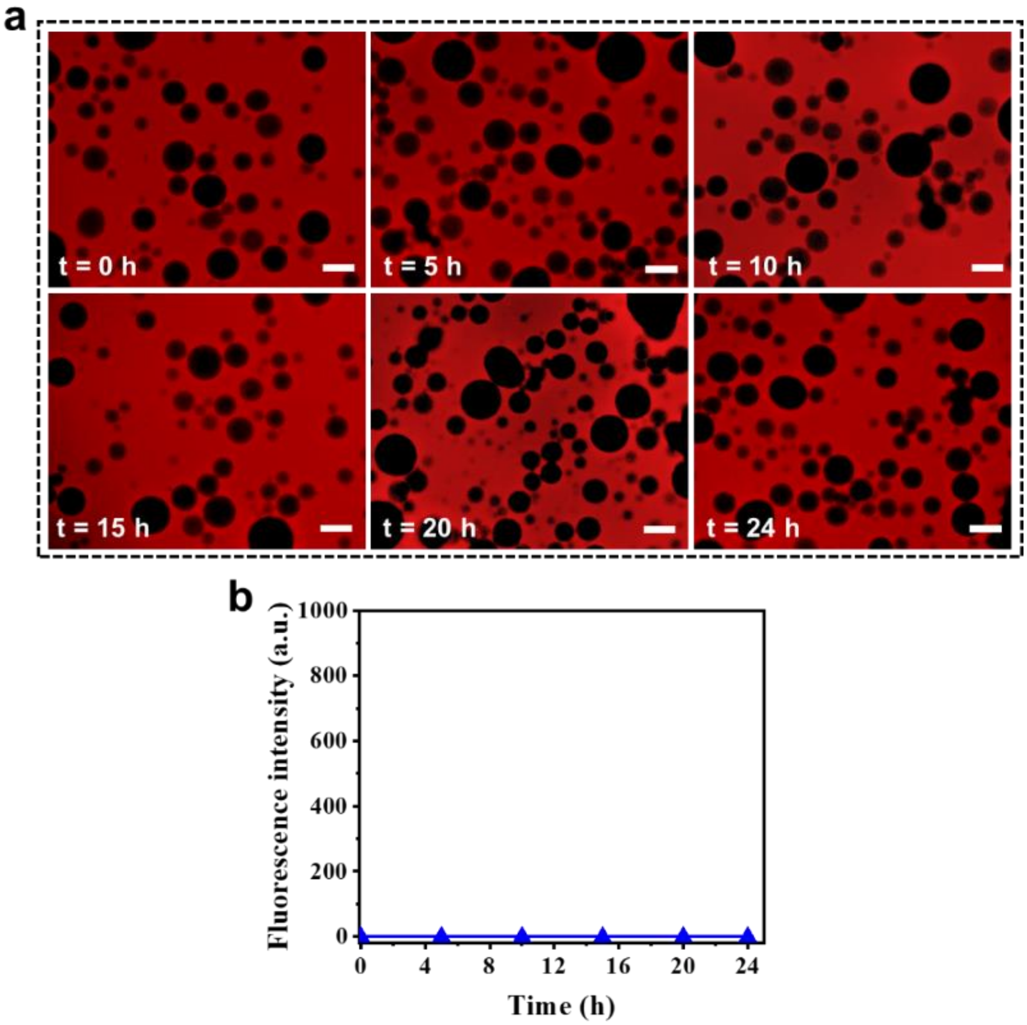

Supplementary Figure 6. Fluorescence microscopy observation of the Pickering double emulsion with outer oil phase dyed by Nile Red. a, Observations as a function of storage time, scale bar = 30 μm. **b**, Fluorescence intensity versus time for the retaining of Nile Red in the outer oil phase.

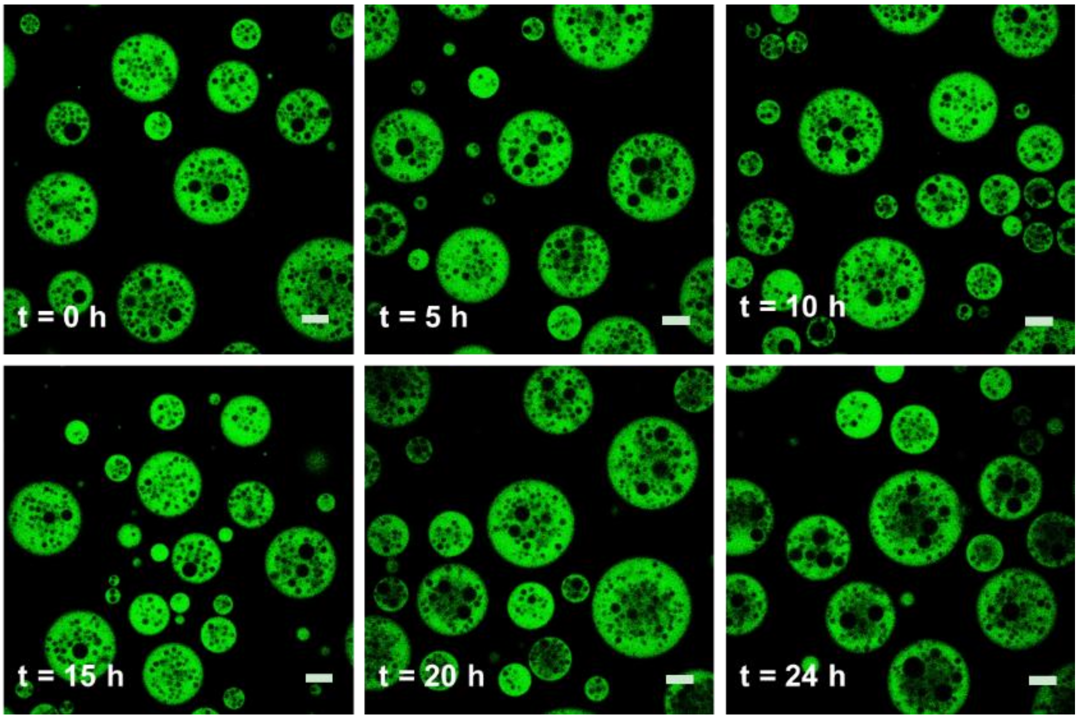

Supplementary Figure 7. Fluorescence microscopy observation of the Pickering double emulsion as a function of time with inner water phase dyed by FITC-Dextran, scale bar = 10 μm.

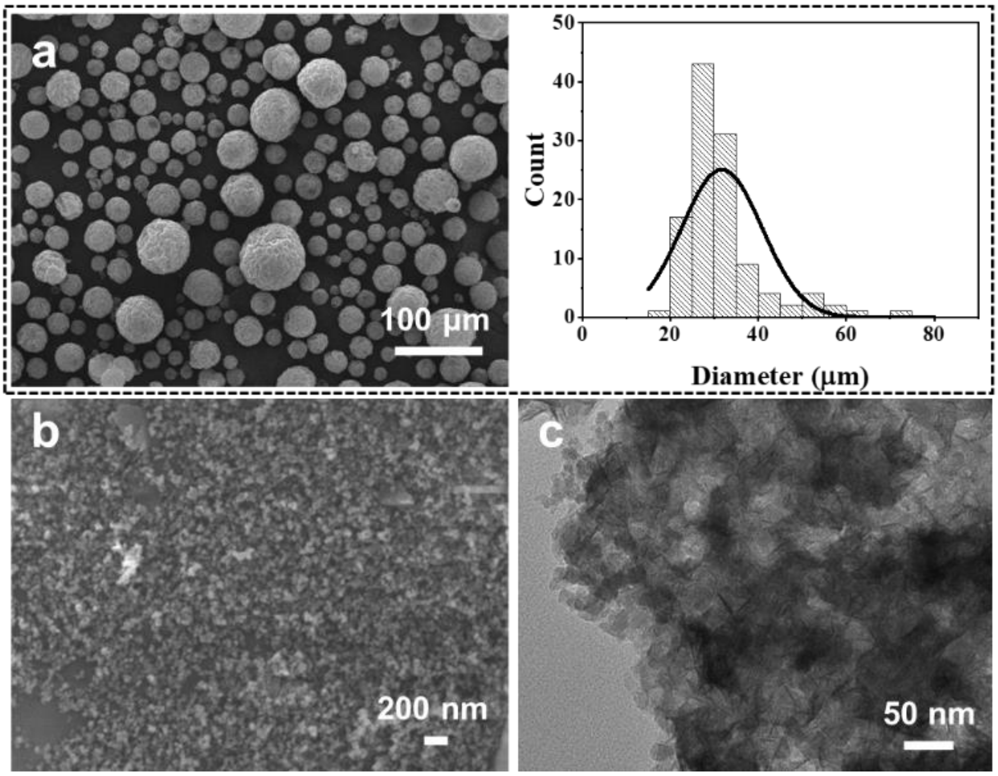

Supplementary Figure 8. Characterization of the multi-compartmental MOF-74 microreactors. **a**, SEM image at low-magnification and its corresponding particle size distribution. **b**, Observation of the silica emulsifier particles on the outer crust. **c**, TEM image of the MOF layer.

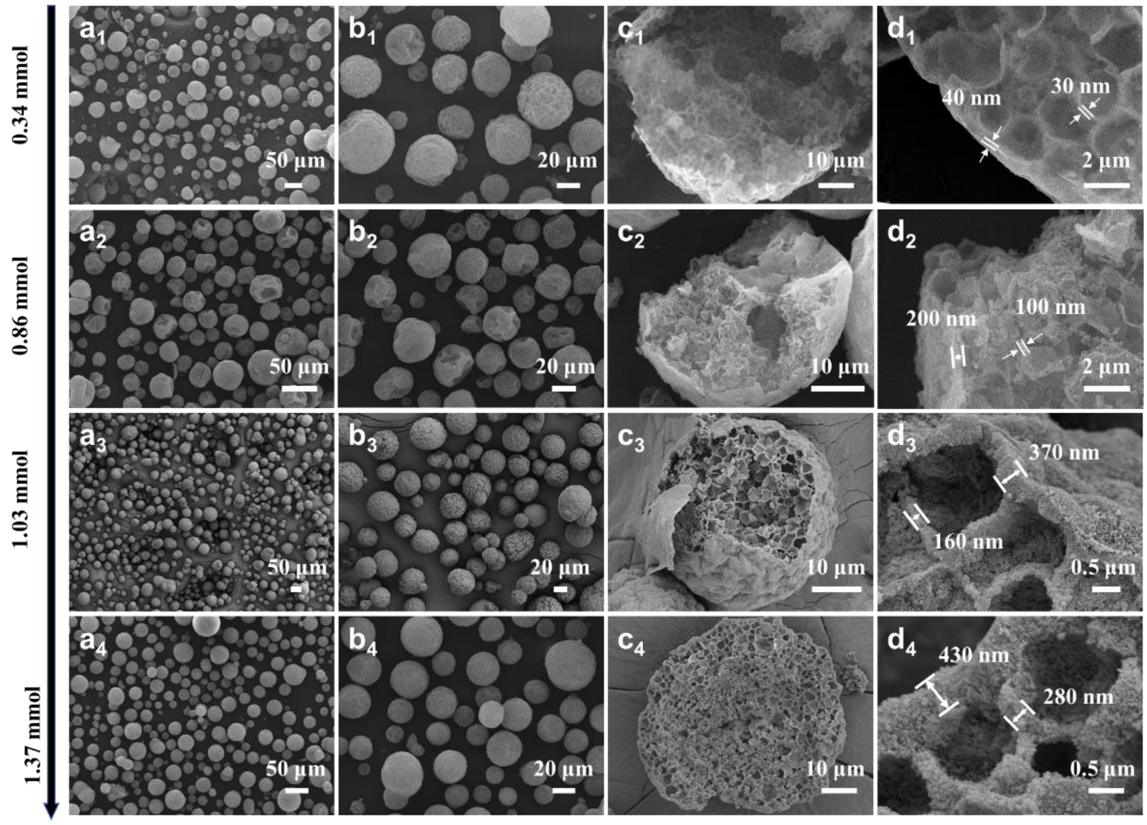

Supplementary Figure 9. SEM images of the multi-compartmental MOF-74 microreactors as a function of metal ion dosage, including 0.34, 0.86, 1.03 and 1.37 mmol. (**a**$_1$–**a**$_4$, **b**$_1$–**b**$_4$) Observations at different magnifications, (**c**$_1$–**c**$_4$) a single broken microparticle, (**d**$_1$–**d**$_4$) high-magnified observation of the interior structures and outer boundary.

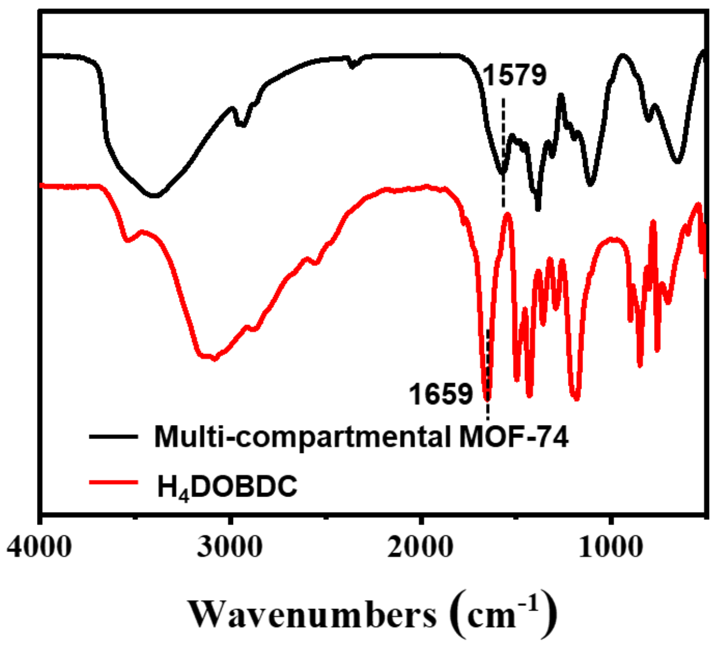

Supplementary Figure 10. FT-IR spectra of the multi-compartmental MOF-74 microreactor and uncoordinated H_4DOBDC ligand. The FT-IR spectra of multi-compartmental MOF-74 microreactor shows the characteristic stretching frequency of carboxylate with a red shift (from 1579 cm^{-1} to 1659 cm^{-1}) compared to uncoordinated H_4DOBDC, confirming the coordination of metal ions and organic ligand.

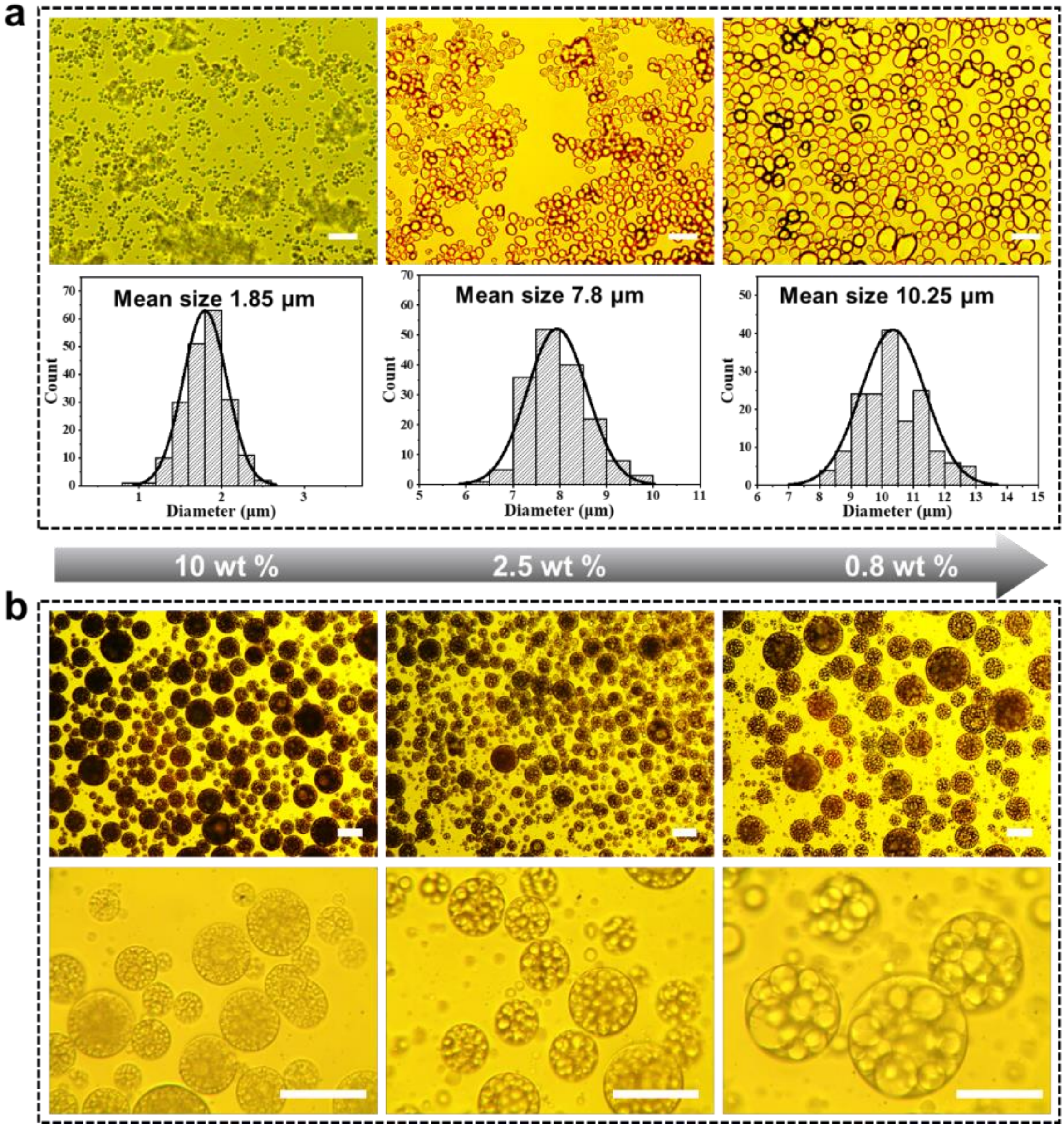

Supplementary Figure 11. Optical micrographs of the primary oil-in-water and final oil-in-water-in-oil double emulsions prepared with different hydrophilic emulsifier dosages (ranging from 10 to 2.5 and 0.8 wt%). **a**, Optical micrographs of the primary oil-in-water emulsion droplets and their size distributions, scale bar= 20 μm. **b**, Optical micrographs of the double emulsions at different magnifications, scale bar= 30 μm.

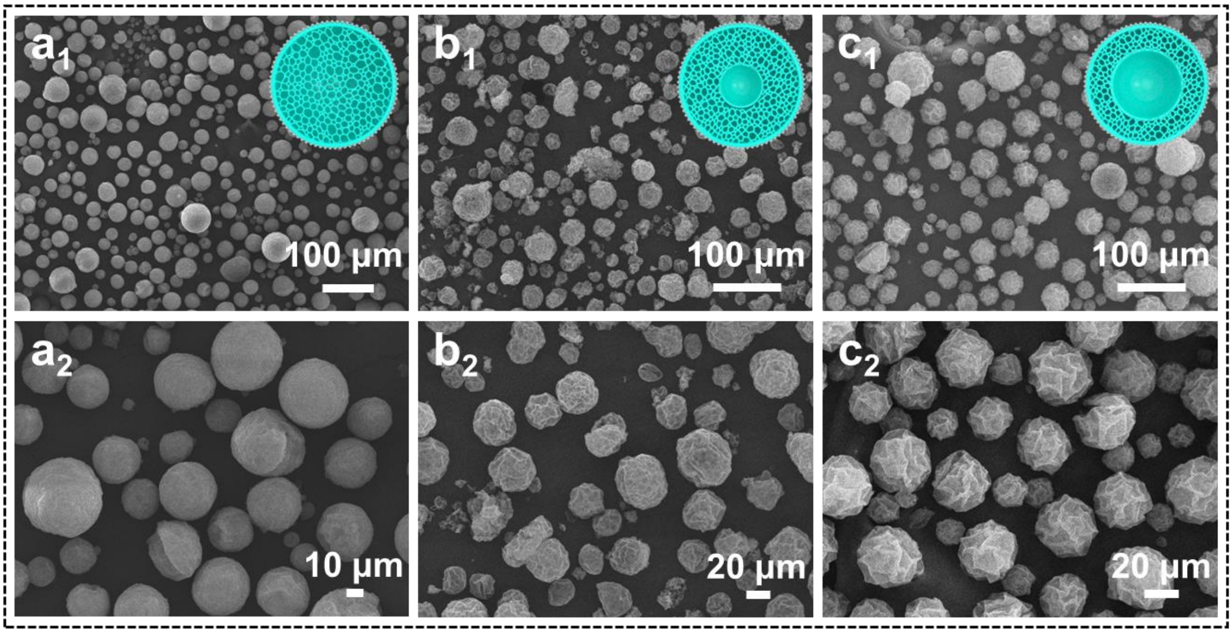

Supplementary Figure 12. SEM images of the multi-compartmental MOF-74 microreactors prepared with different inner droplet volume fractions. (**a1, a2**) 80%, (**b1, b2**) 70%, (**c1, c2**) 65%.

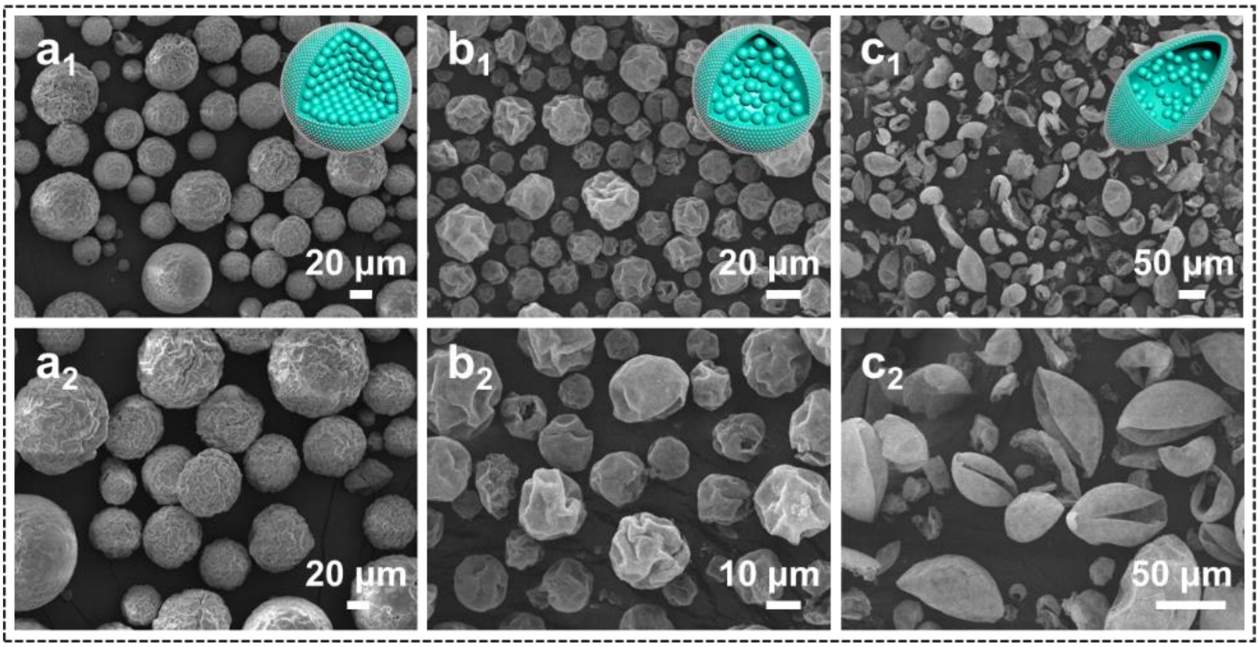

Supplementary Figure 13. SEM images of the multi-compartmental MOF-74 microreactors prepared with different inner droplet volume fractions by an additional pre-coordination process. (**a$_1$, a$_2$**) 80%, (**b$_1$, b$_2$**) 70%, (**c$_1$, c$_2$**) 65%.

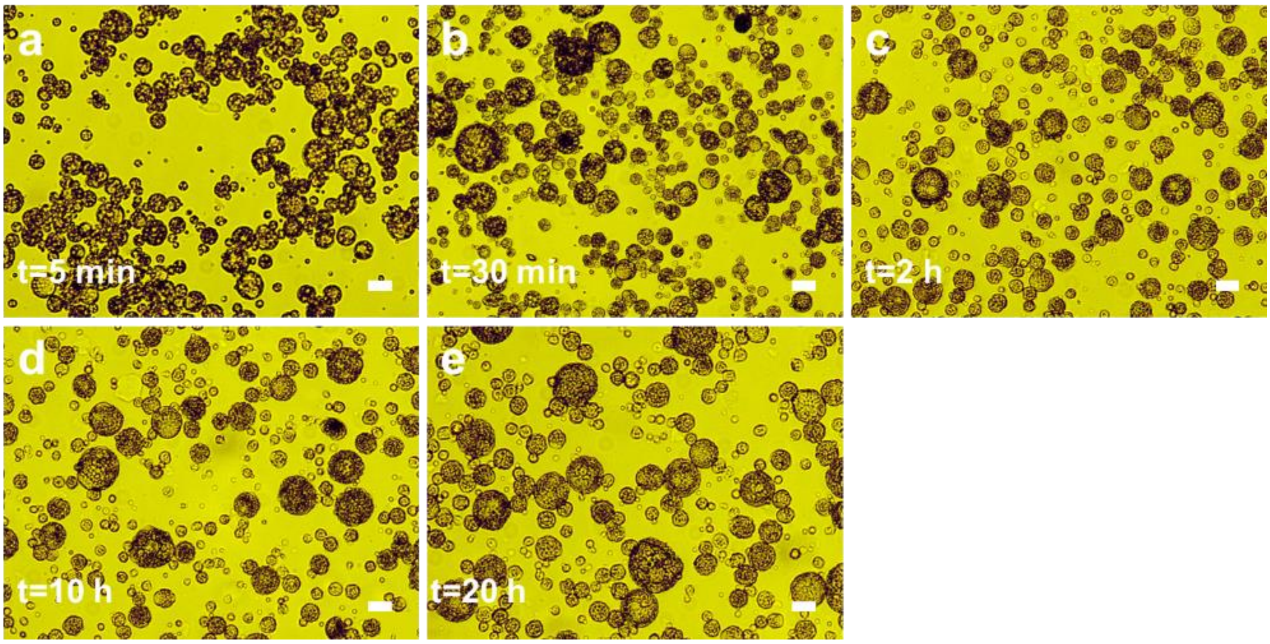

Supplementary Figure 14. Optical microscopy observations for the formation of multi-compartmental MOF-74 microreactor at different growth times (after growth for a desired time, the sample was isolated and dispersed into ethanol for observation). **a**, 5 min. **b**, 30 min. **c**, 2 h. **d**, 10 h. **e**, 20 h. Scale bar = 30 μm.

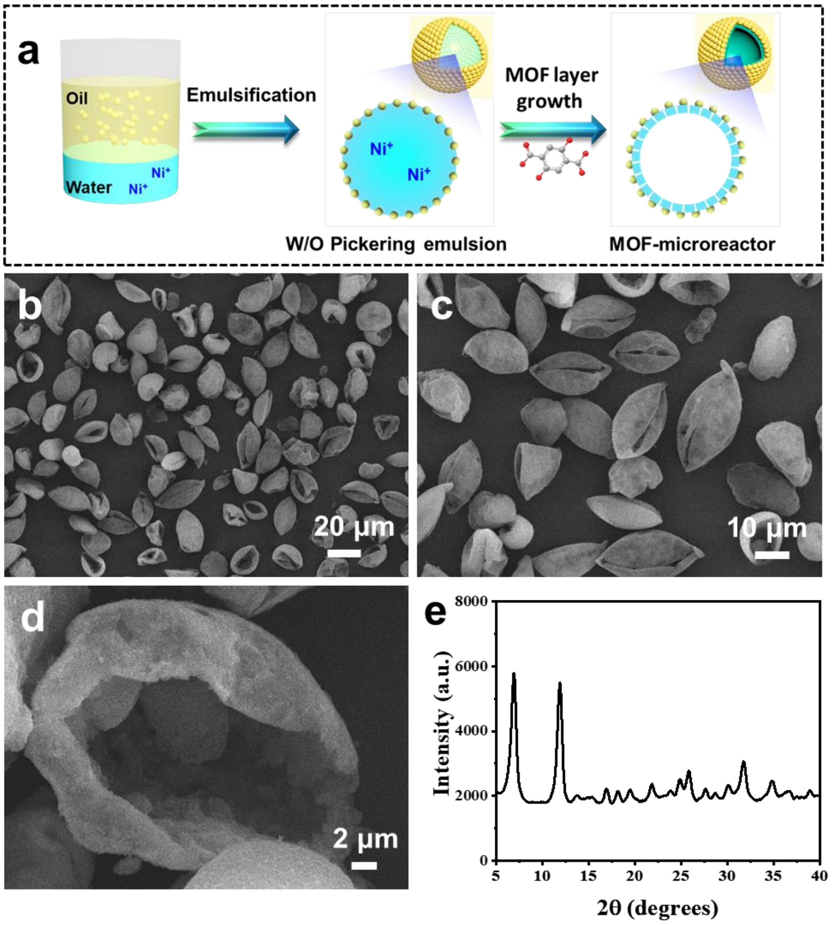

Supplementary Figure 15. Characterization of the single compartment MOF-74 that derived from water-in-oil Pickering emulsions. a, Schematic illustration of the synthesis process. **b**, SEM observation at low magnification. **c**, The magnified observation. **d**, A single broken microcapsule revealing the hollow interior. **e**, XRD pattern.

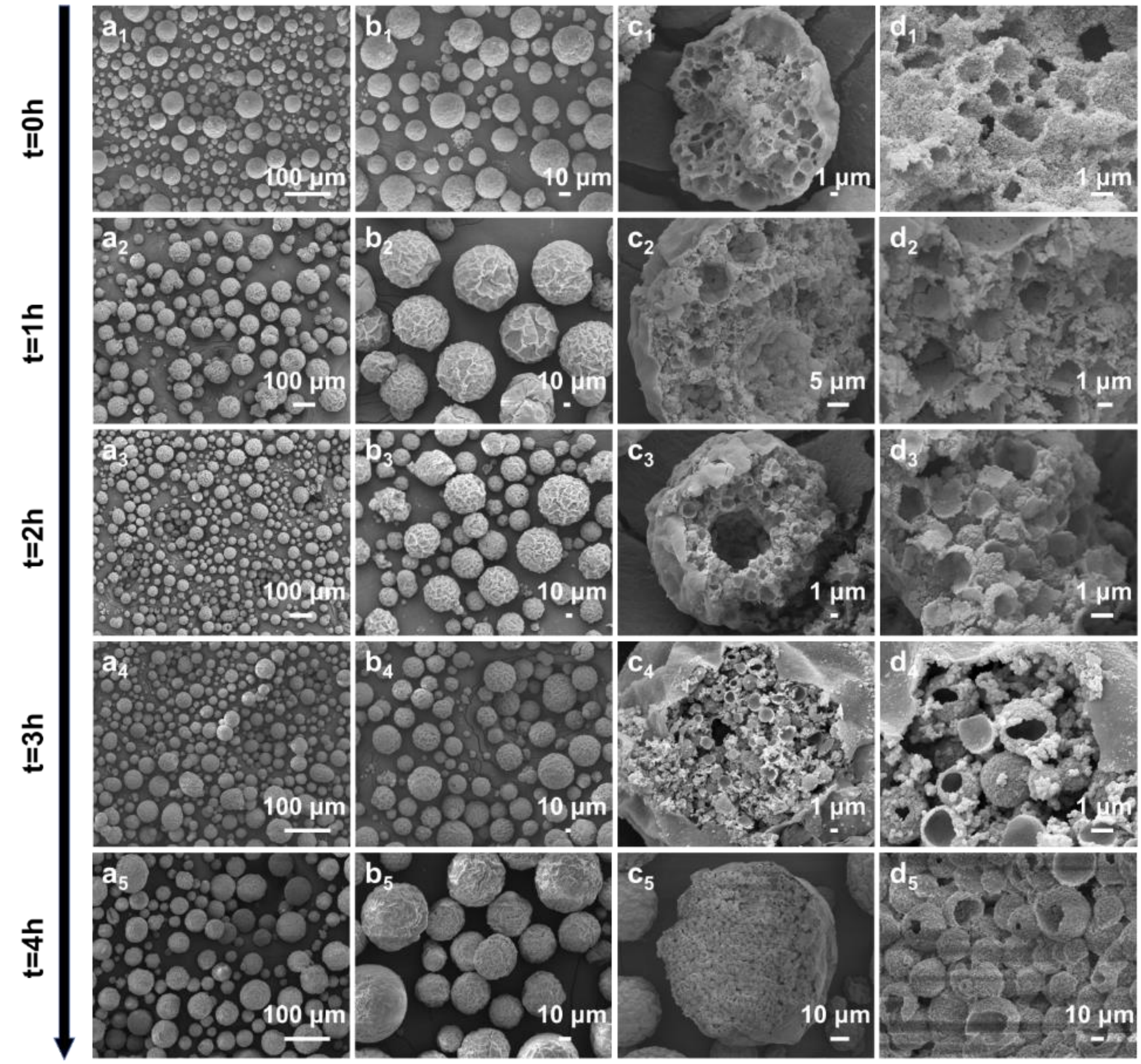

Supplementary Figure 16. SEM images of the multi-compartmental MOF-74 microreactors as a function of pre-coordination time. (**a_1–a_5, b_1–b_5**) Observations at different magnifications, (**c_1–c_5**) a single broken microparticle, (**d_1–d_5**) high-magnified observation of the interior structures.

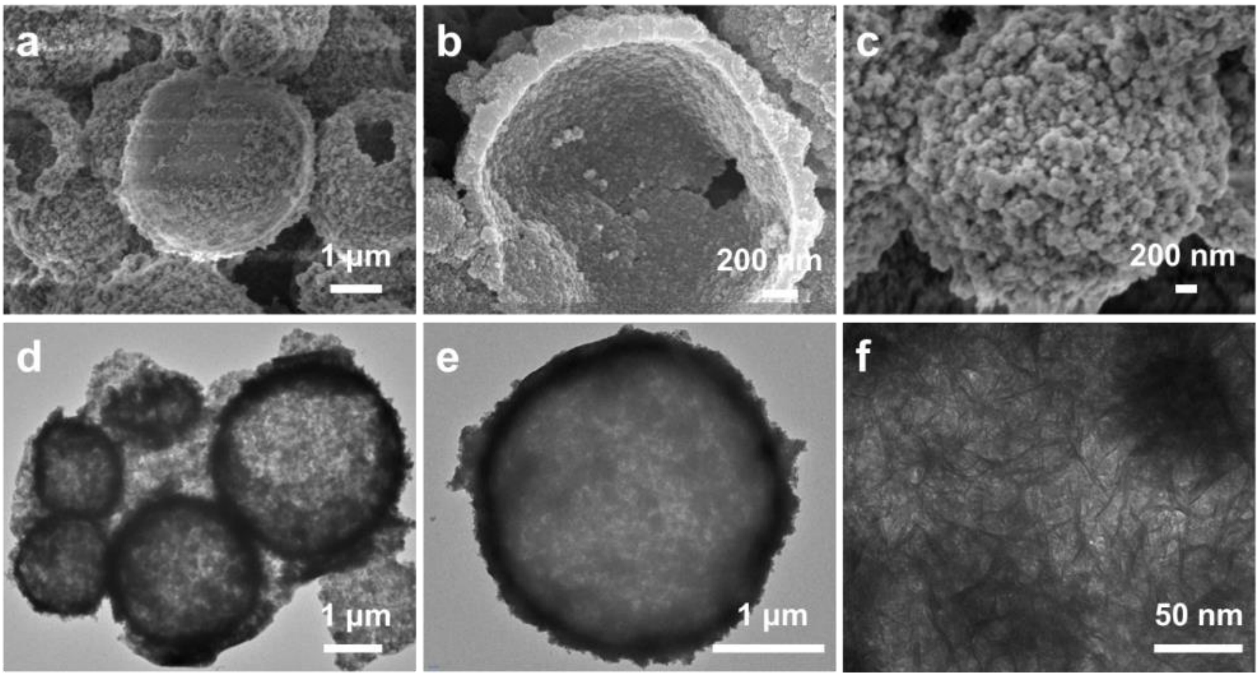

Supplementary Figure 17. Observation of the interior compartments of MOF-74 microreactors that prepared with a pre-coordination process (4 h). **a**, SEM image at low magnification, **b**, a single broken particle showing the hollow structure with a dense MOF layer, **c**, an intact particle, (**d, e**) TEM images of the hollow microstructures, **f**, a magnified observation of the dense MOF layer.

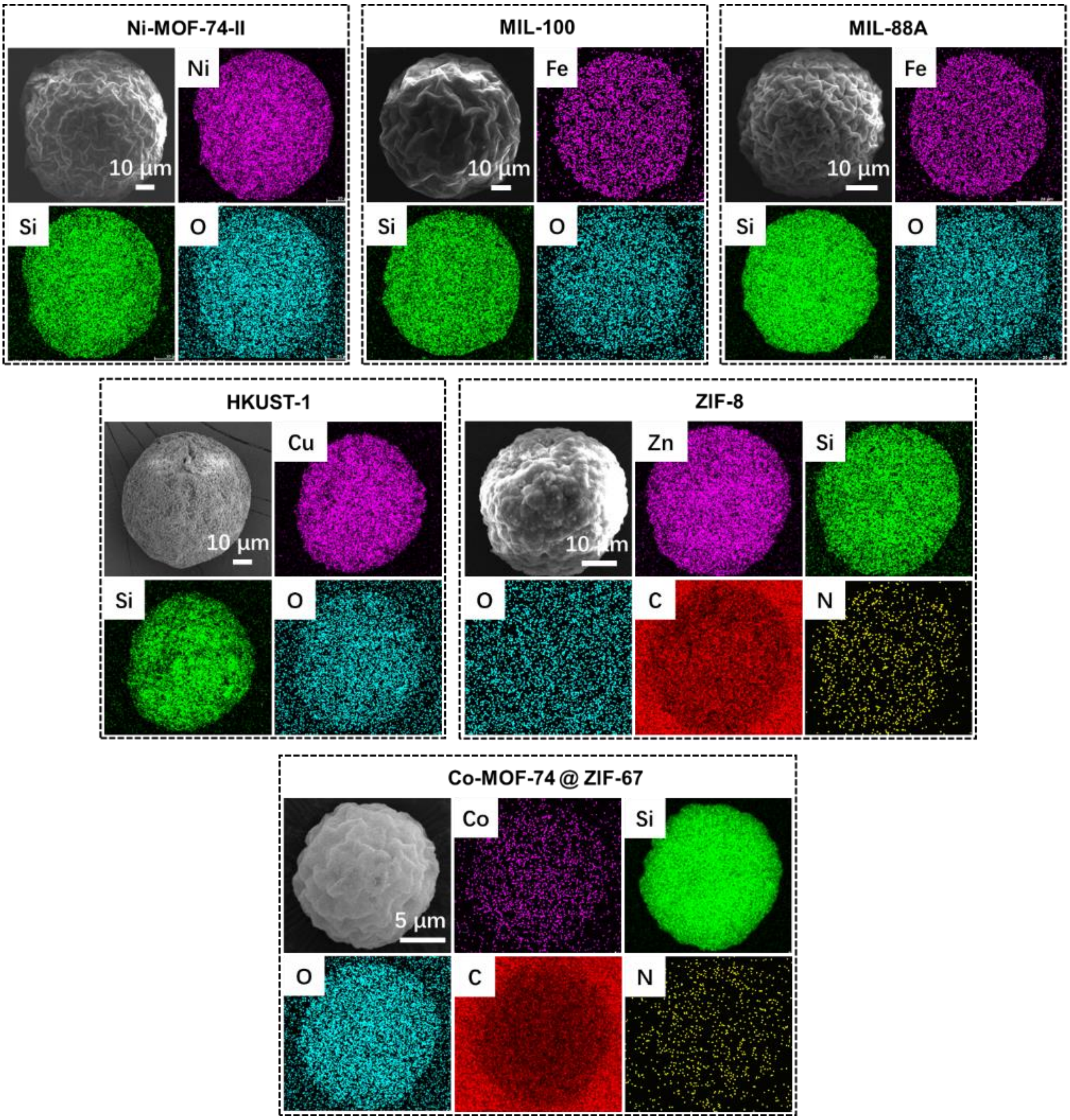

Supplementary Figure 18. Elemental mappings of various multi-compartmental MOF microreactors, including Ni-MOF-74-II, MIL-100, MIL-88A, HKUST-1, ZIF-8, and Co-MOF-74@ZIF-67.

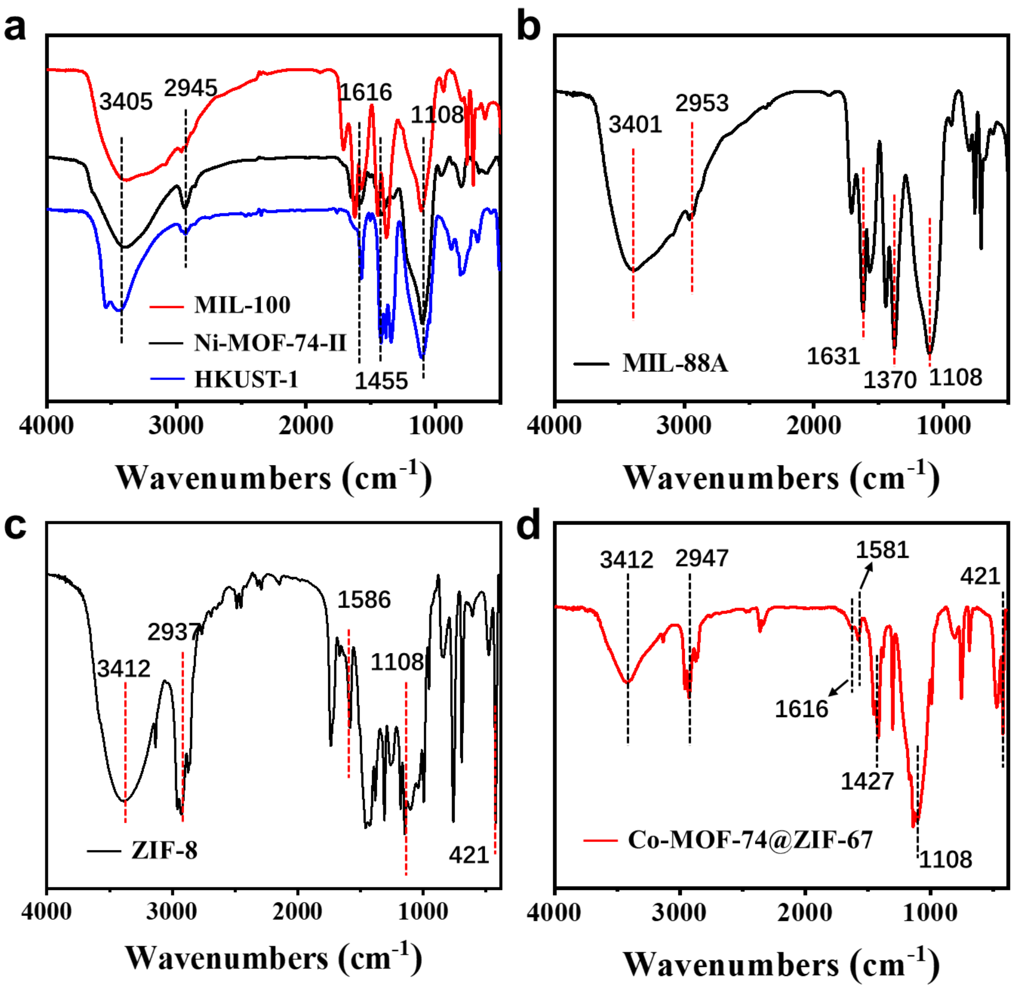

Supplementary Figure 19. FT-IR spectra of various multi-compartmental MOF microreactors. **a**, Ni-MOF-74-II, MIL-100 and HKUST-1. **b**, MIL-88A. **c**, ZIF-8. **d**, Co-MOF-74@ZIF-67.

Notes: a. The observed bands at 1616 and 1455 cm^{-1} are associated with benzene rings of Ni-MOF-74-II, MIL-100 or HKUST-1, the bands at 2945 cm^{-1} are assigned to C-H of grafted $-CH_3$ groups, the peaks at 1108 and 3405 cm^{-1} are characteristic bands of Si-O, O-H in silica emulsifier.

b, The bands at 1370 and 1631 cm^{-1} are respectively from symmetric and asymmetric vibration modes of the carboxyl group, the bands at 2951 cm^{-1} are assigned to C-H of grafted $-CH_3$ groups, the peaks at 1108 and 3401 cm^{-1} are characteristic bands of Si-O, O-H in silica emulsifier.

c, The characteristic adsorption bands of ZIF-8 were observed at 1586 and 421 cm^{-1}, ascribed to C = N stretching vibration on the imidazole ring and Zn-N stretching vibration respectively. The bands at 2937 cm^{-1} are assigned to C-H of grafted $-CH_3$ groups, the peaks at 1108 and 3412 cm^{-1} are characteristic bands of Si-O, O-H in silica emulsifier.

d, The peaks at 1581 and 421 cm^{-1} are respectively assigned to C = N stretching vibration on the imidazole ring and Zn-N stretching vibration of ZIF-67. The observed bands at 1616 and 1427 cm^{-1} are all associated with benzene rings of MOF-74. The bands at 2947 cm^{-1} are assigned to C-H of grafted $-CH_3$ groups, the peaks at 1108 and 3412 cm^{-1} are characteristic bands of Si-O, O-H in silica emulsifier.

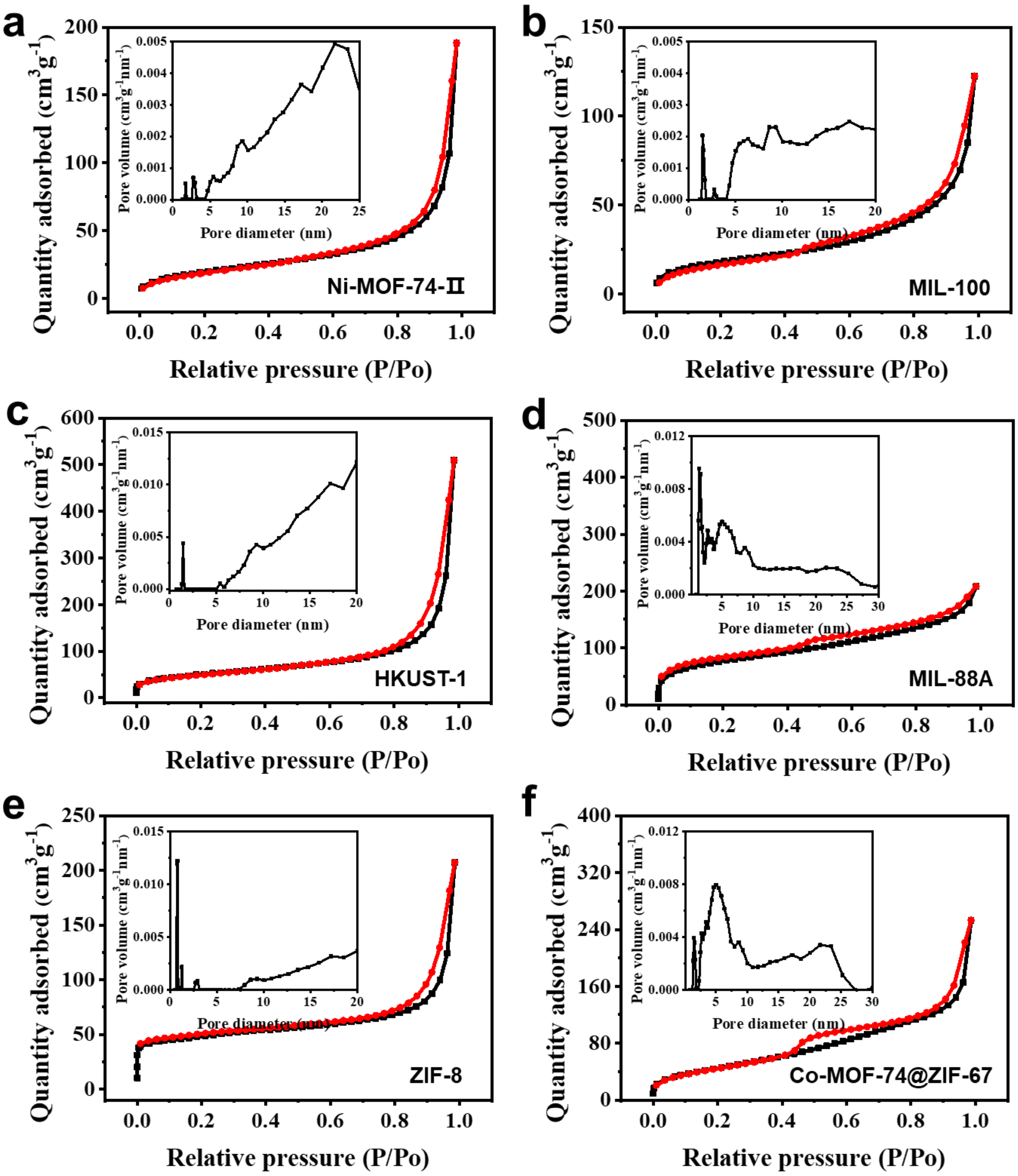

Supplementary Figure 20. N_2 sorption characterization of various multi-compartmental MOF microreactors. **a**, Ni-MOF-74-II. **b**, MIL-100. **c**, HKUST-1. **d**, MIL-88A. **e**, ZIF-8. **f**, Co-MOF-74@ZIF-67, insets are pore size distributions from DFT method.

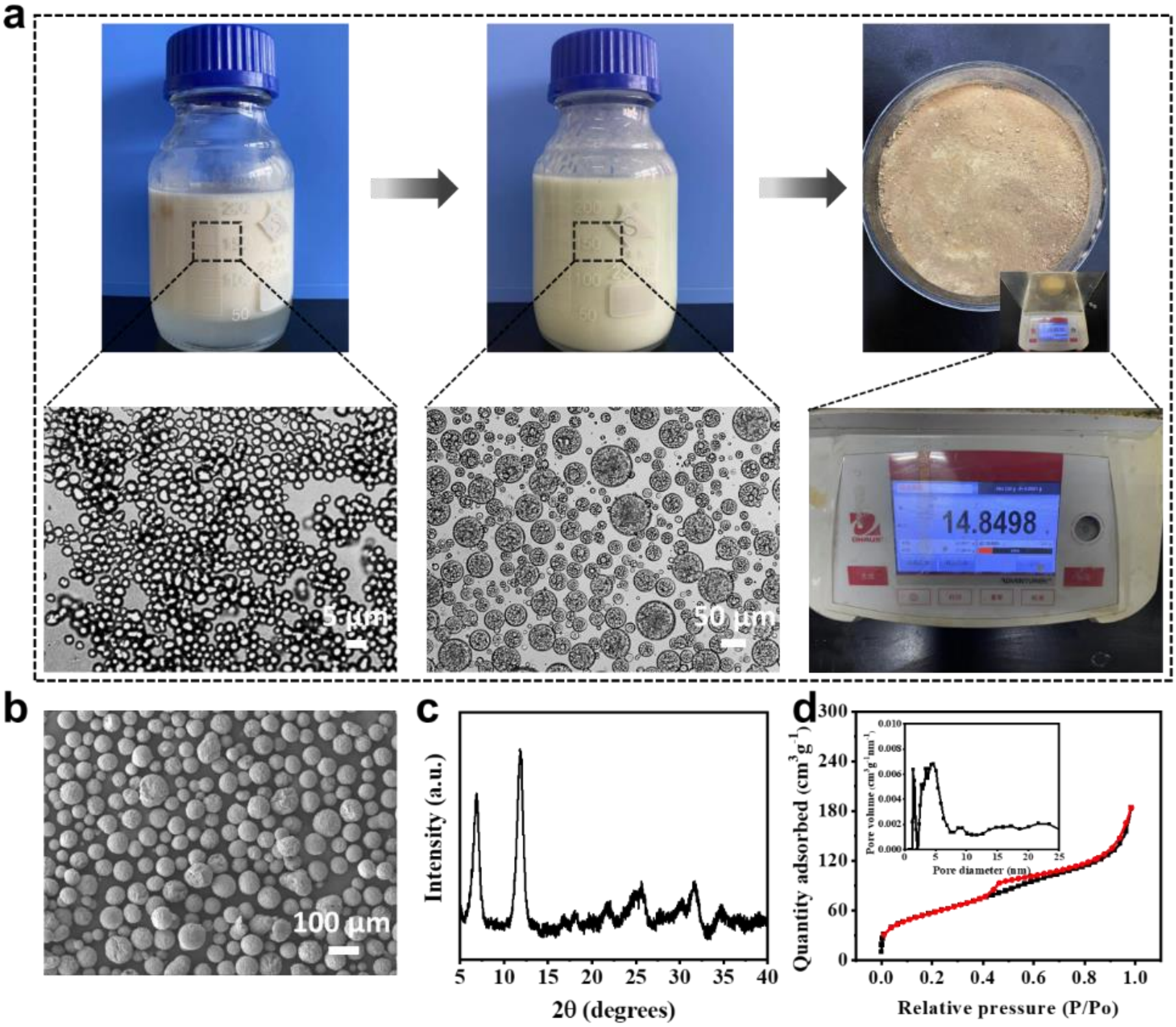

Supplementary Figure 21. Large-scale production of multi-compartmental MOF-74 microreactors (250 mL Pickering double emulsion with 14.8 g of products). **a**, Digital images and optical micrographs of the scaled emulsions and their derived materials. **b**, SEM image. **c**, XRD pattern. **d**, N_2 sorption characterization.

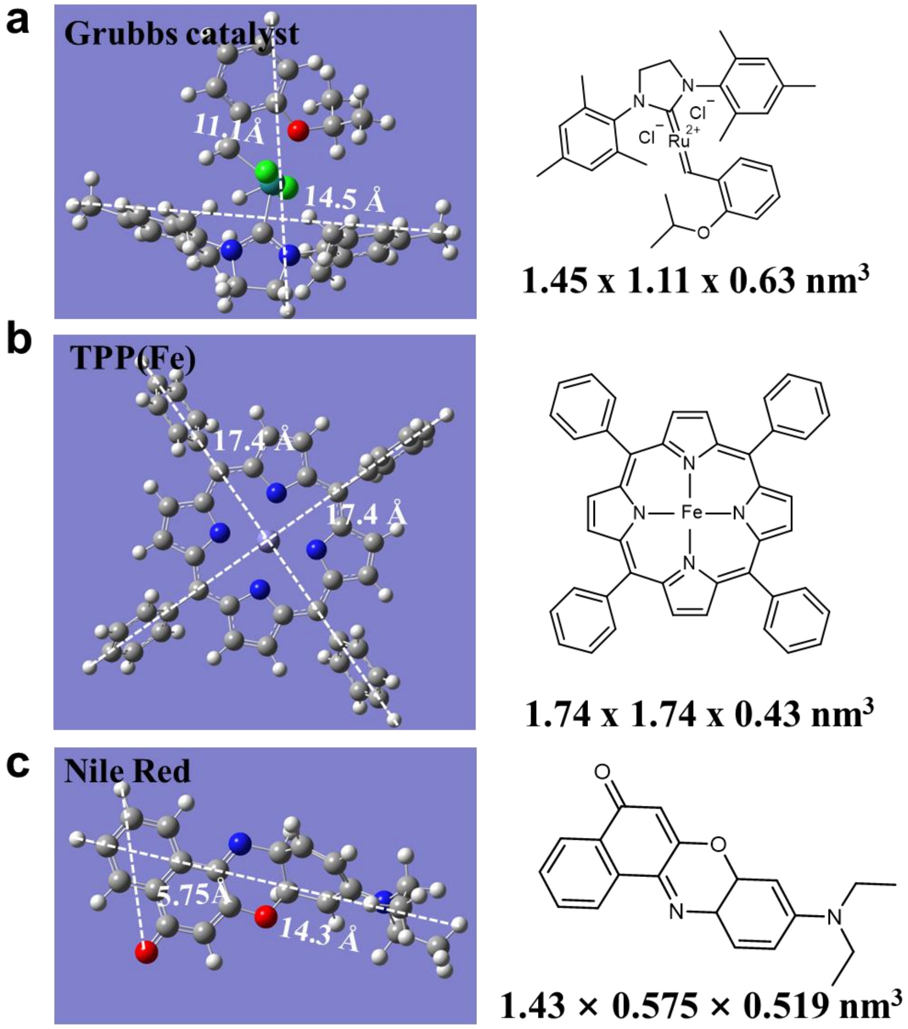

Supplementary Figure 22. Molecular structure and size of the probe molecules. a, Grubbs catalyst. **b**, TPP(Fe). **c**, Nile Red.

Notes: The optimized molecular structures for Grubbs' catalyst, TPP(Fe), and Nile Red were obtained using the Gaussian 09 software of D01 revision. Specifically, For Grubbs catalyst molecule, we employed the B3LYP (functional) and LANL2DZ (basis set). As for the other molecules, we used the B3LYP (functional) and 6-31g(d) (basis set) for the calculations.

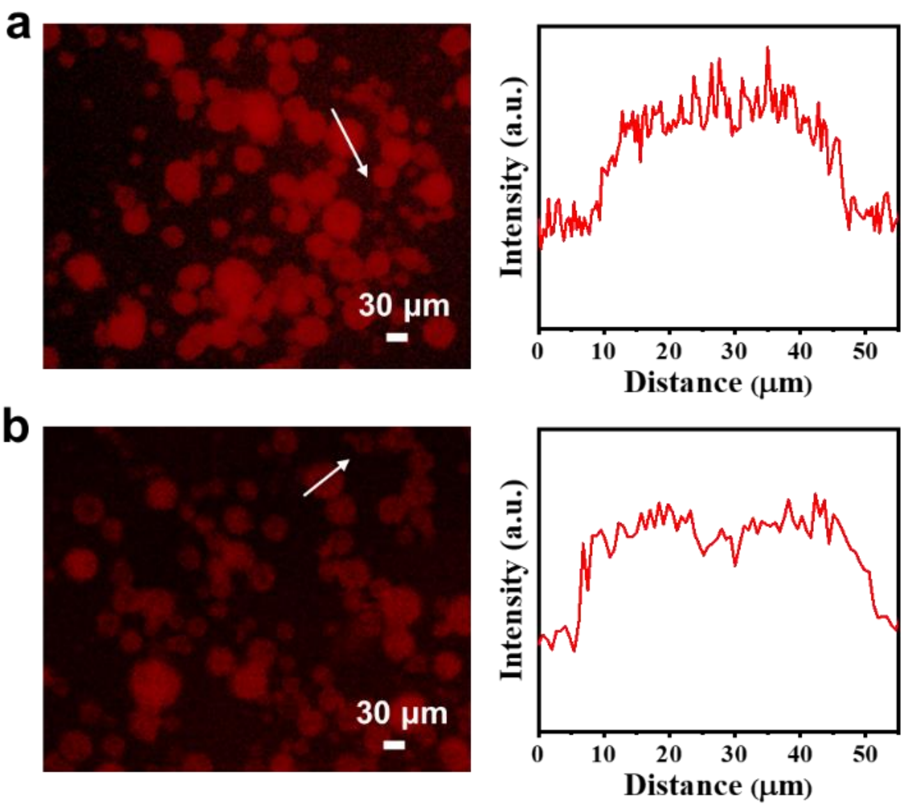

Supplementary Figure 23. Confocal fluorescence microscopes and their corresponding intensity profiles of the multi-compartmental MOF-74 microreactors loaded with Rhodamine B-labelled enzymes. a, GOx. **b**, HRP.

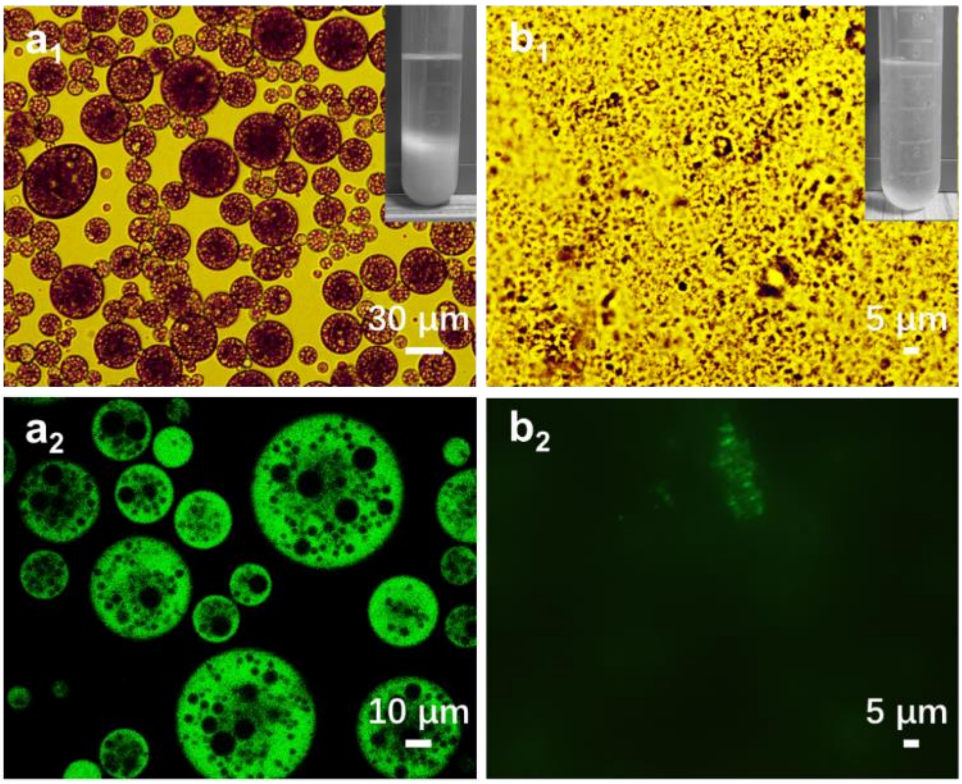

Supplementary Figure 24. Stability tests of the enzymatic Pickering double emulsion in polar solvent. (**a**$_1$, **b**$_1$) Optical micrographs of the emulsion droplets before and after exposure to ethanol, (**a**$_2$, **b**$_2$) confocal fluorescence microscopy observations of the emulsion droplets before and after exposure to ethanol (confined with FITC-Dextran-labeled CALB).

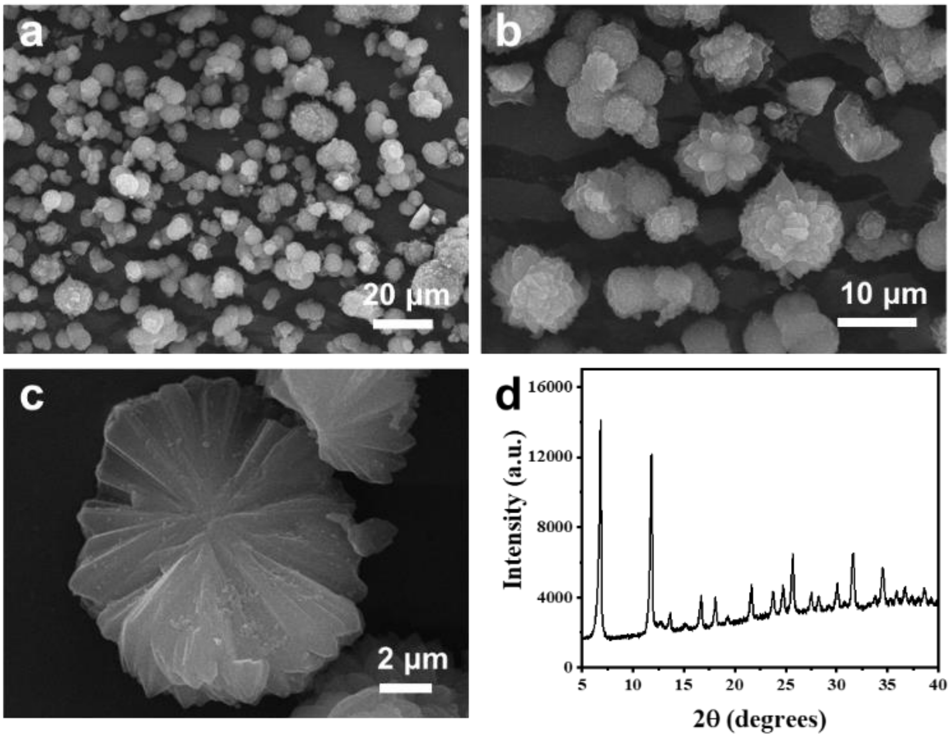

Supplementary Figure 25. Characterization of the MOF-74 material synthesized by traditional solvothermal method. **a**, Representative SEM images at low magnification. **b**, A larger magnification observation. **c**, A single broken particle. **d**, XRD pattern.

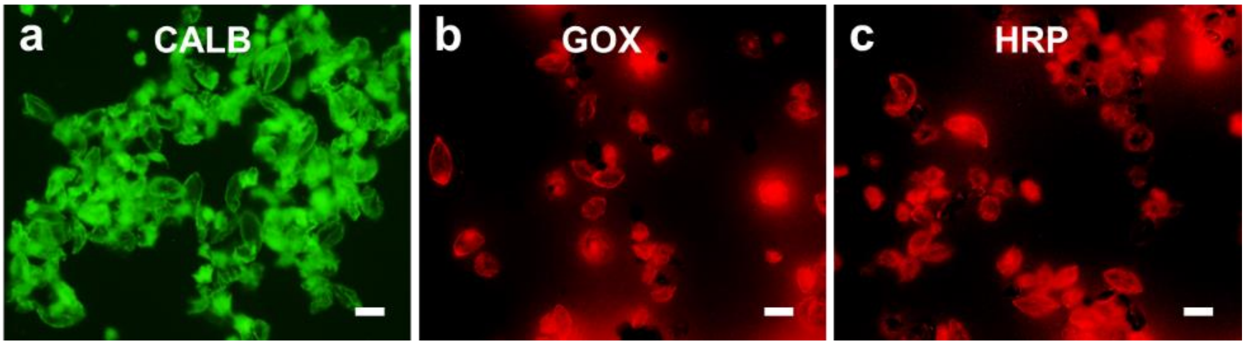

Supplementary Figure 26. Confocal fluorescence microscopy of the single compartment MOF-74 microreactor encapsulated with different enzymes. **a**, FITC-labelled CALB. **b**, Rhodamine B-labelled GOx. **c**, Rhodamine B-labelled HRP, scale bar = 20 μm.

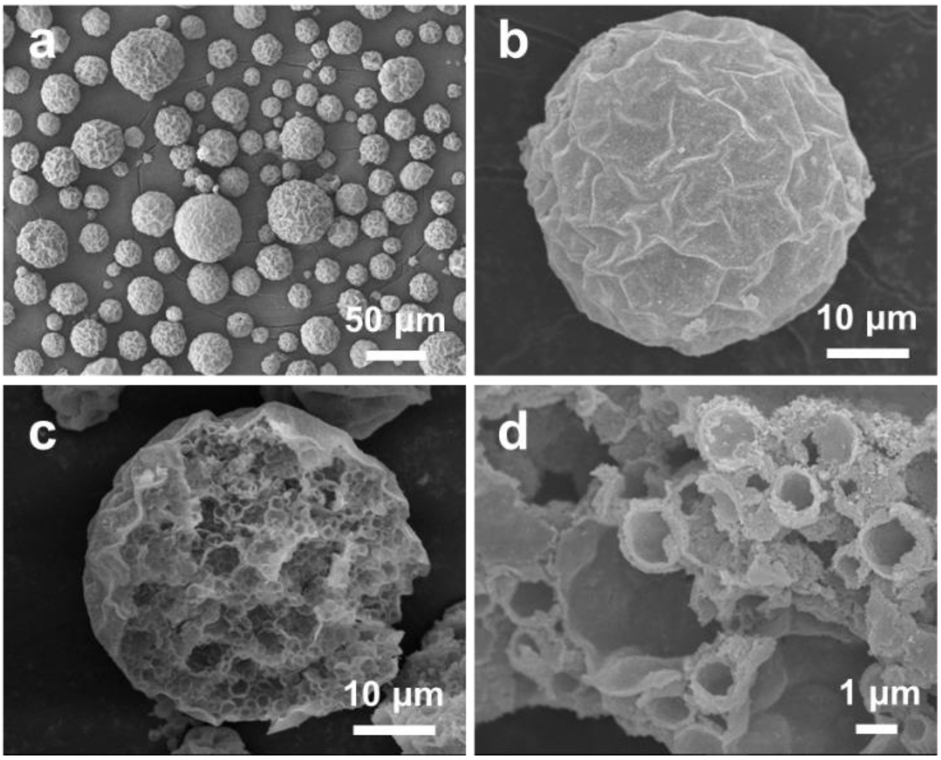

Supplementary Figure 27. SEM images of the multi-compartmental MOF-74 microreactor after loading with Grubbs catalyst and CALB. a, Low magnification observation. **b**, A single particle. **c**, A broken microsphere. **d**, A magnified observation of the interior structure.

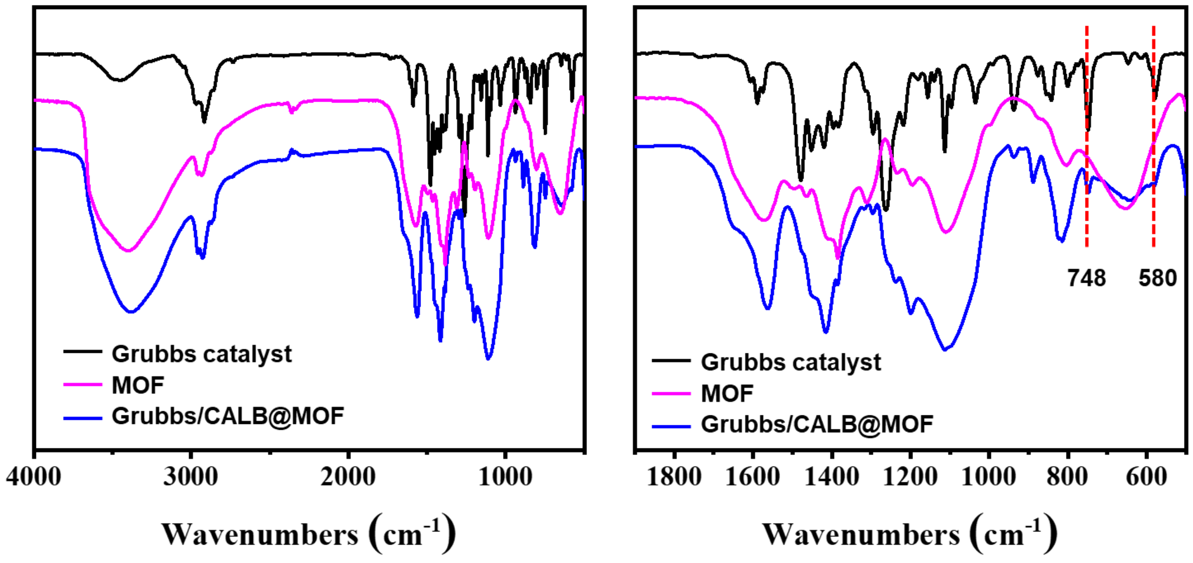

Supplementary Figure 28. FT-IR spectra of Grubbs catalyst, the multi-compartmental MOF-74 and Grubbs/CALB@MOF-74 solid catalyst. Right spectrum is local magnification in the range of 2000-500 cm^{-1}.

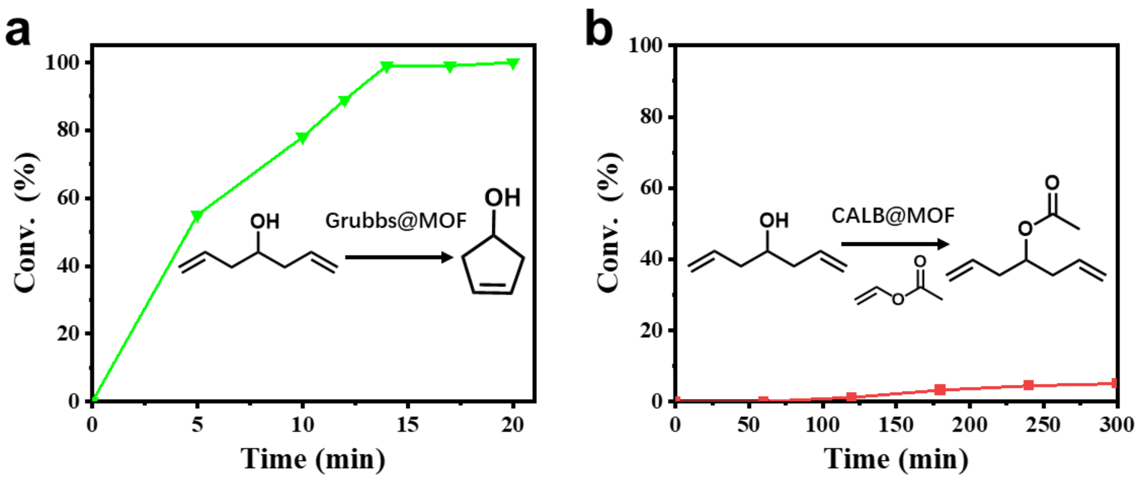

Supplementary Figure 29. Kinetic plots for the two catalytic steps over separated solid catalysts. **a**, Ring-closing metathesis of 1,6-heptadien-4-ol over Grubbs@MOF. **b**, Transesterification reaction of 1,6-heptadien-4-ol over CALB@MOF.

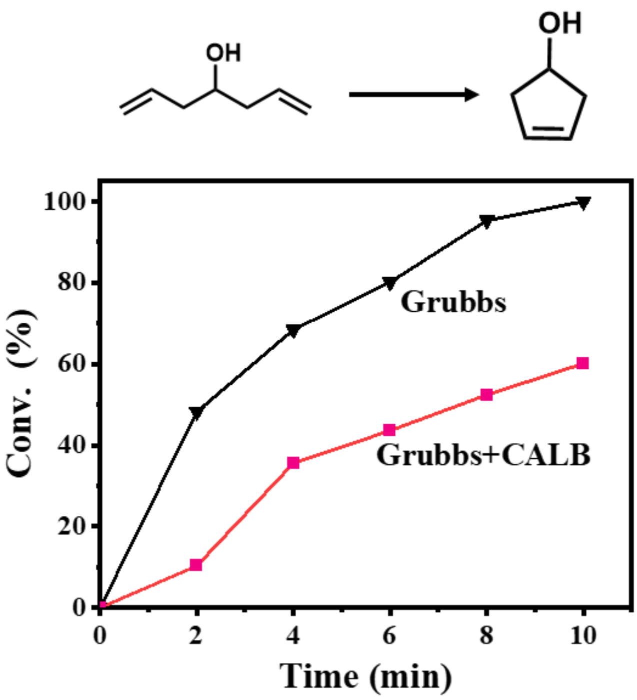

Supplementary Figure 30. Kinetic plots for ring-closing metathesis of 1,6-heptadien-4-ol over the homogenous mixture of Grubbs/CALB and pure Grubbs' catalyst. Reaction conditions: 0.1 mmol 1,6-heptadien-4-ol, 1 mL *n*-hexane, 5 mg Grubbs' catalyst or the mixture of 5 mg Grubbs' catalyst and 0.24 mg enzyme, 45 ºC.

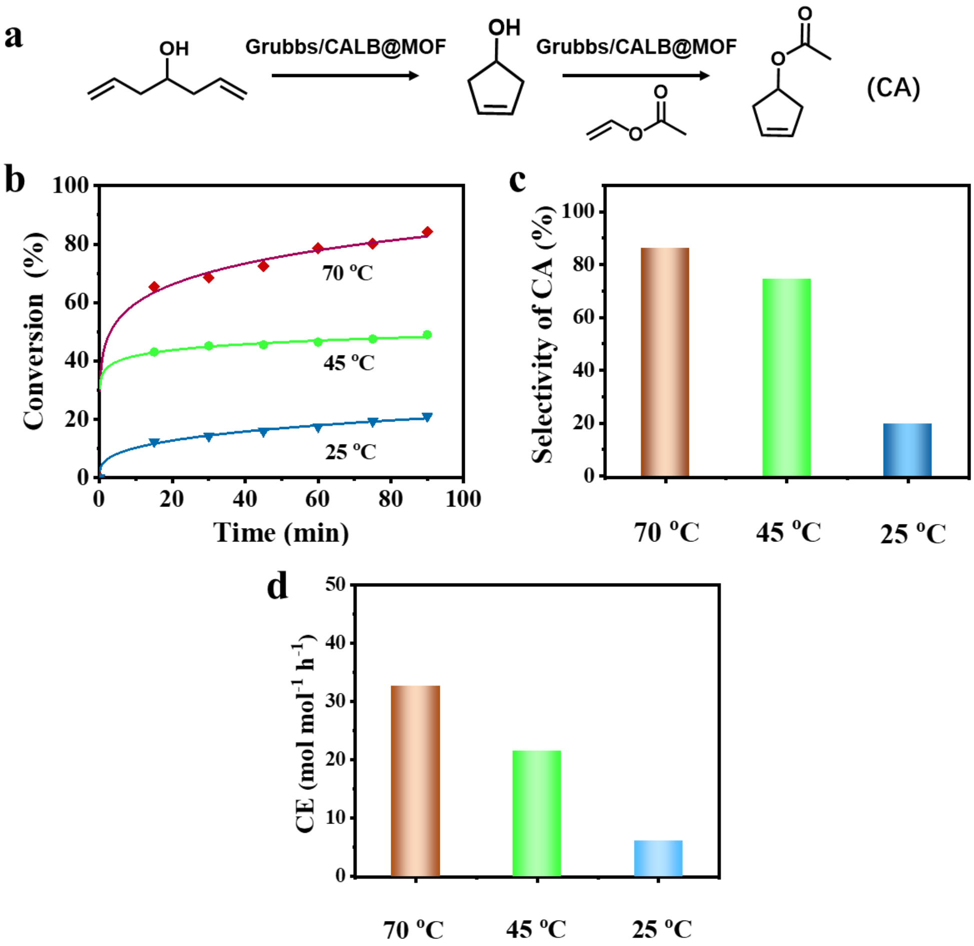

Supplementary Figure 31. Catalytic results of Grubbs/CALB@MOF microreactors for the ring-closing metathesis/transesterification cascade reaction at different temperatures. a, Reaction networks. **b,** Kinetic plots. **c,** Selectivity of aimed CA product. **d,** Calculated catalysis efficiency.

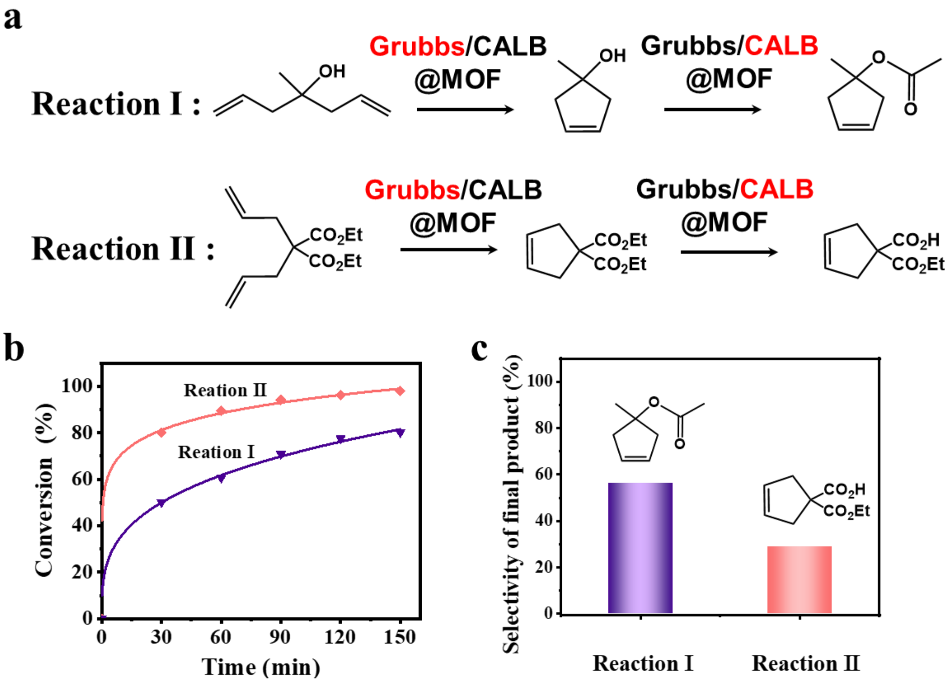

Supplementary Figure 32. Catalytic performance of Grubbs/CALB@MOF in the one-pot cascade reaction of different substrates. a, Reaction networks. **b**, Kinetic plots. **c**, Selectivity of aimed product.

Note: Reaction conditions: For reaction I, 0.1 mmol 4-methyl-1,6-heptadien-4-ol, 1 mL dichloromethane, 200 mg Grubbs/CALB@MOF (the loading content of CALB and Grubbs' catalyst were 8 mg g^{-1} and 25 mg g^{-1} respectively), 60 ºC. For reaction II, 0.1 mmol diethyl diallylmalonate, 1 mL n-hexane, 300 μL deionized water, 200 mg Grubbs/CALB@MOF (the loading content of CALB and Grubbs' catalyst were 8 mg g^{-1} and 25 mg g^{-1} respectively), 45 ºC.

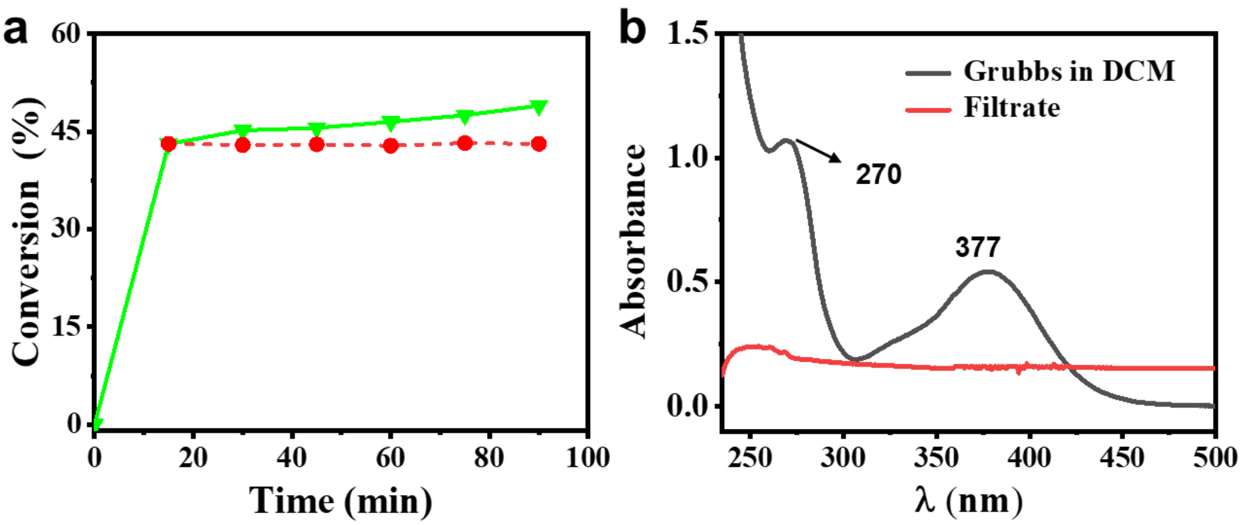

Supplementary Figure 33. The stability and recyclability test of Grubbs/CALB@MOF catalyst. **a**, Kinetic profiles of the chemo-enzymatic cascade reaction over Grubbs/CALB@MOF-74 and the filtrate reaction. **b**, UV/Vis spectra of the filtrate after reaction and homogeneous Grubbs' catalyst in dichloromethane (DCM).

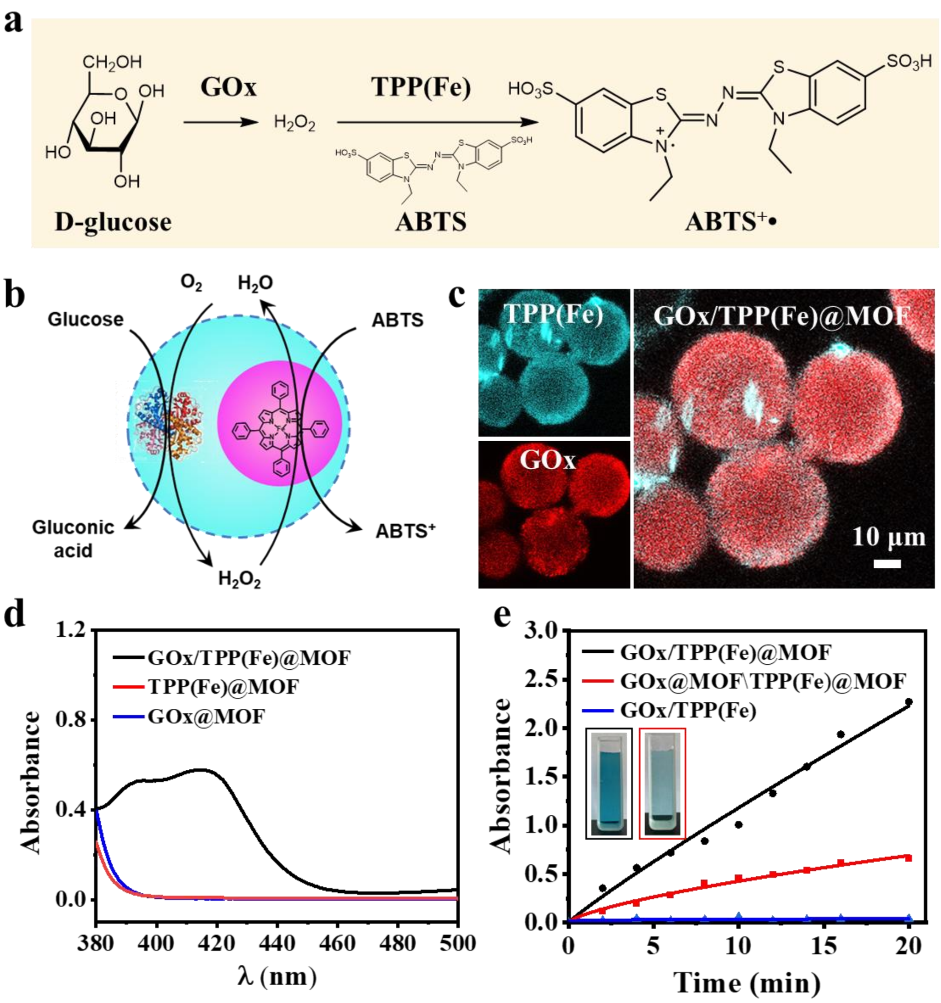

Supplementary Figure 34. Chemo-enzymatic cascade catalysis in glucose oxidase (GOx)/Fe-porphyrin driven oxidation reaction. a, Reaction equation. **b**, Schematic showing the one-pot cascade situation. **c**, Confocal fluorescence microscopy images of GOx/TPP(Fe)@MOF (blue for TPP(Fe) and red for Rhodamine B-labelled GOx). **d**, UV-Vis absorption spectra of the reaction solution of GOx/TPP(Fe)@MOF, GOx@MOF and TPP(Fe)@MOF. **e**, Time-dependent absorbance changes of the oxidation product in different catalytic systems, including GOx/TPP(Fe)@MOF, mechanically mixed system (GOx@MOF/TPP(Fe)@MOF) and homogeneous counterpart (GOx/TPP(Fe)).

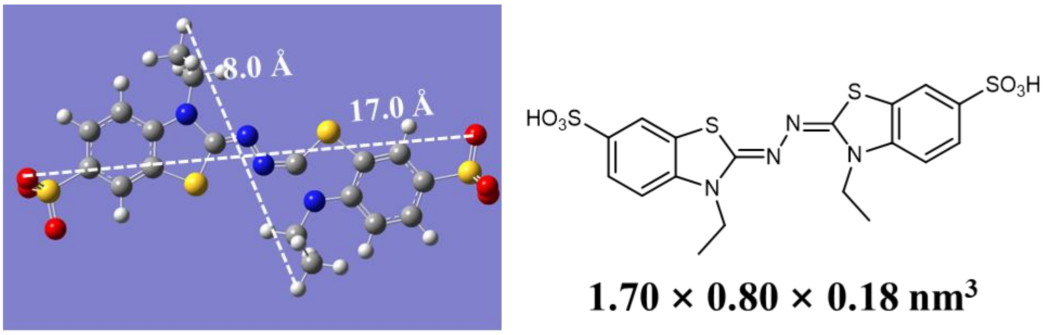

Supplementary Figure 35. Molecular structure and size of ABTS. The optimized molecular structure for ABTS is obtained by Gaussian 09 software at the B3LYP/6-31g(d) level.

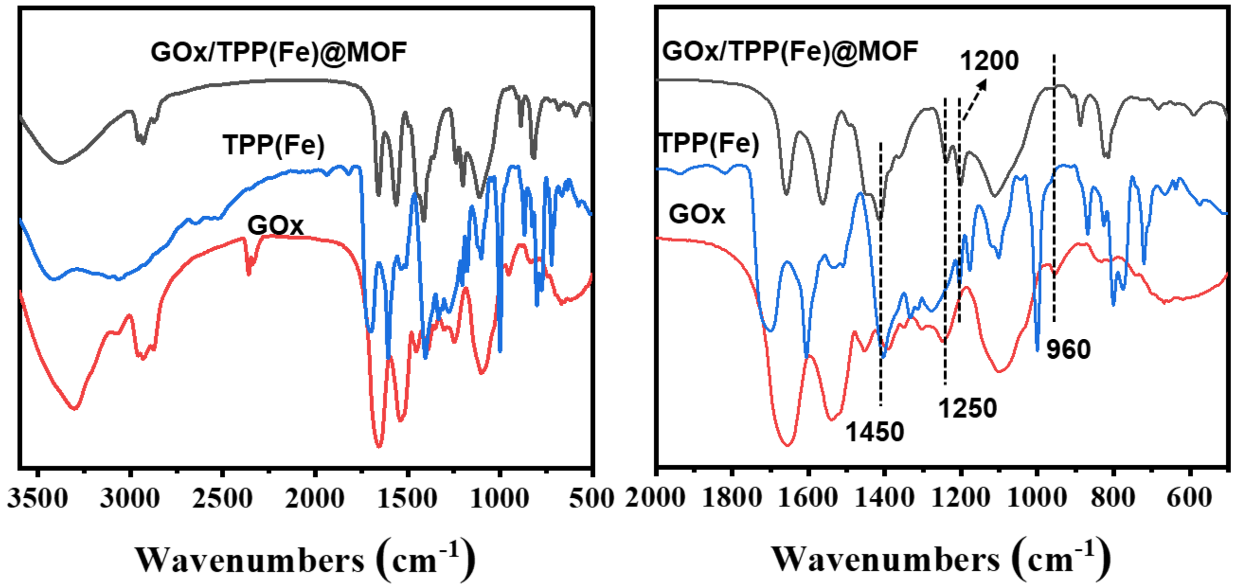

Supplementary Figure 36. FT-IR spectra of GOx/TPP(Fe)@MOF-74, GOx and TPP(Fe). Right spectrum is local magnification in the range of 2000-500 cm^{-1}.

Note: The characteristic absorption bands in the range from ~2870 to 2970 cm^{-1} belong to the methyl or methylene peak of glucose oxidase. The peaks at 960 and 1250 cm^{-1} are ascribed to the imino group of GOx.

The peaks at 1200 cm^{-1} and 1500-1600 cm^{-1} are corresponding to the porphyrin ring vibrations. The bands at 1450 cm^{-1} are associated with $\nu_{C\text{-}N}$ of the porphyrin ring.

Supplementary Table 1. Metal salts and organic ligands of the synthesized multi-compartmental MOF microreactors and their corresponding textural properties.

Entry	Sample	Metal salt	Organic linker	S ($m^2\ g^{-1}$)	V ($cm^3\ g^{-1}$)
**1**	MOF-74	$Ni(NO_3)\bullet 6H_2O$	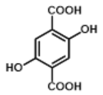	166.6	0.22
**2**	Ni-MOF-74-II	$Ni(NO_3)\bullet 6H_2O$	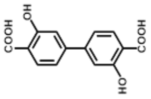	72.8	0.29
**3**	MIL-100	$FeCl_3\bullet 6H_2O$	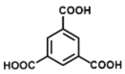	66.2	0.19
**4**	MIL-88A	$FeCl_3\bullet 6H_2O$	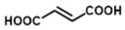	274.5	0.32
**5**	HKUST-1	$Cu(NO_3)_2\bullet 3H_2O$	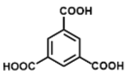	179.1	0.79
**6**	ZIF-8	$Zn(NO_3)_2\bullet 6H_2O$	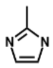	164.0	0.32
**7**	Co-MOF-74@ZIF-67	$Co(OAc)_2\bullet 4H_2O$	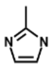 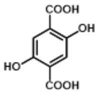	167.7	0.39

Supplementary Table 2. Encapsulation efficiencies of various enzymes and molecular catalysts in different systems.

Systems / Probe species	Pickering double emulsion	Traditional MOF-74	Single compartment MOF-74	Multi-compartmental MOF-74
**CALB**	0.8%	18%	99.0%	95.8%
**GOx**	1.3%	21%	99.0%	96.1%
**HRP**	2.7%	10%	99.0%	97.0%
**Grubbs' catalyst**	0%	18.7%	0%	77.9%
**TPP(Fe)**	0%	16.8%	0%	79.4%

Supplementary Methods

1. Chemicals and Reagents

All chemicals and reagents were used as received without any further purification. Fumed silica N20 (20 nm) was purchased from Wacker Chemie. $Ni(NO_3)_2{\cdot}6H_2O$, $FeCl_3{\cdot}6H_2O$, $Cu(NO_3)_2{\cdot}3H_2O$, $Zn(NO_3)_2{\cdot}6H_2O$, $Co(OAc)_2{\bullet}4H_2O$, *n*-octanol (AR), *n*-hexane (AR), toluene and tributylamine were purchased from Shanghai Chemical Reagent Company of the Chinese Medicine Group. Methyltrimethoxysilane (98%), dimethyldichlorosilane, *n*-hexylamine (99%), triethylamine (TEA, 99%), rhodamine B (99%), fluorescein isothiocyanate Dextran (FITC-Dextran, CAS No. 60842-46-8), fluorescein isothiocyanate isomer I (FITC-I, CAS No. 3326-32-7), poly(vinyl alcohol) (PVA), 2,5-dihydroxyterephthalic acid, 4,4'-dihydroxybiphenyl-3,3'-dicarboxylic acid, 1,3,5-benzenetricarboxylic acid (H_3BTC), fumaric, 2-methylimidazole, Grubbs catalyst (CAS No. 301224-40-8), 1,6-heptadien-4-ol (98%) and vinyl acetate (99%) were purchased from Aladdin (China). Nile red (CAS No. 7385-67-3), iron tetraphenylporphyrin chloride (TPP(Fe), CAS No. 16456-81-8), glucose oxidase (GOx), peroxidase (HRP) and 2,2'-azino-bis(3-ethylbenzothiazoline-6-sulfonic acid (ABTS) were obtained from Sigma-Aldrich. Native lipase B from Candida Antarctica (CALB) was purchased from Novozymes. The water used in this study was de-ionized water.

2. Characterization

Emulsion droplets were observed using an optical microscope analyzer (XSP-8CA, Shanghai, China) equipped with 10 or 40 × magnification lens. Confocal laser scanning microscopy images were obtained on a Carl Zeiss LSM880 instrument (Germany), the excitation wavelengths of FITC-I, Nile red and Rhodamine B are 488 nm (green), 530 nm (red) and 554 nm (red) respectively. The contact angles of water in air were measured on a Krüss DSA100 instrument. Before measurement, the powder sample was compressed into a disk of thickness approximately 1 mm (*ca.* 5 MPa). Thermogravimetric analysis (TGA) was performed under an air atmosphere with a heating rate of 5 °C min^{-1} by using a SDT Q600 thermogravimetric analyzer. FT-IR spectra were collected with a Bruker Tensor II spectrometer in the range 400–4000 cm^{-1}. Scanning electron microscope (SEM) images and EDS elemental maps were performed using a Hitachi SU 1510. Nitrogen-sorption analysis was performed at –196 °C on a Micromeritics ASAP 2020 analyzer. Before measurement, samples were degassed at 120 °C under vacuum for 6 h. The specific surface area was calculated from the adsorption branch in

the relative pressure range of 0.05–0.25 using the Brunauer-Emmett-Teller (BET) method. The total pore volume was estimated from the amount adsorbed at the P/P_0 value of 0.99. Pore size distributions were determined from DFT method. Samples for transmission electron microscopy (TEM) observation were prepared by dispersing the sample powder in ethanol using ultrasound and then allowing a drop of the suspension to evaporate on a copper grid covered with a holey carbon film. TEM images were obtained on a JEOL-JEM-2000EX instrument. The powder X-ray diffraction data were collected on a Rigaku D/Max2500PC diffractometer with Cu Kα radiation (λ = 1.5406 Å) over the 2θ range of 2–40° with a scan speed of 2 °C min^{-1} at room temperature. UV-Vis determination was conducted on a TU-1900 spectrometer (China). The content of elemental ruthenium or iron was determined using an Agilent 720ES inductively coupled plasma optical emission spectrometer (ICP-OES). Gas chromatography (GC) analysis was carried out on an Agilent 7890 analyzer (HP-5) with a flame ionization detector.

3. Theoretical Calculations

The optimized molecular structures for Grubbs' catalyst, TPP(Fe), Nile Red and ABTS were obtained using the Gaussian 09 software of D01 revision. Specifically, for Grubbs' catalyst molecule, we employed the B3LYP (functional) and LANL2DZ (basis set). As for the other molecules, we used the B3LYP (functional) and 6-31g(d) (basis set) for the calculations.

4. Material Synthesis

Synthesis of hydrophilic emulsifier. Commercial silica nanoparticles (1.0 g, dried at 120 °C for 4 h before use) were dispersed into 30 mL toluene under sonication, followed by addition of 0.25 mmol $(MeO)_3SiCH_3$ and 0.5 mmol Et_3N (as catalyst). The mixture was stirred under an N_2 atmosphere at 120 °C for 4 h. Then, the solid product was isolated by centrifugation, washed with toluene and dried under vacuum, yielding hydrophilic silica emulsifier.

Synthesis of hydrophobic emulsifier. Commercial silica nanoparticles (1.0 g, dried at 120 °C for 4 h before use) were dispersed into 30 mL toluene under sonication. Then, 3 mmol $(CH_3)_2SiCl_2$ and 6 mmol $CH_3(CH_2)_5NH_2$ (as catalyst) were added into this suspension. The mixture was stirred under a N_2 atmosphere at 60 °C for 4 h. The solid particles were collected through centrifugation, washed with toluene and dried, yielding hydrophobic silica emulsifier.

Synthesis of fluorescently labelled silica emulsifiers. The hydrophilic and hydrophobic emulsifiers were labelled with fluorescein isothiocyanate isomer I (FITC-I) and Rhodamine B respectively. Specifically, 1.0 g of hydrophilic silica emulsifier together with 0.001 mmol 3-aminopropyltriethoxysilane (APTES) were dispersed into 8 mL toluene. After stirring for 2 h under a N_2 atmosphere at 60 °C, the solid material was isolated by centrifugation, washed with toluene and dried, yielding amino-functionalized silica. Then, this sample was re-dispersed into 50 mL ethanol, and added with 10 mg FITC-I. The mixture was stirred overnight in the dark at room temperature. After centrifugation, the solid was collected and throughly washed with ethanol, yielding FITC-I-labelled silica. As for the Rhodamine B-labelled hydrophobic silica emulsifier, a similar method was used.

Synthesis of fluorescently labelled enzymes. CALB and GOx (or HRP) were labelled with FITC-Dextran and Rhodamine B, respectively. Specifically, native CALB (2.0 mL) and FITC-Dextran solution (2×10^{-3} M, 200 μL) were added into a PBS solution (phosphate buffer: 0.05 M Na_2HPO_4–0.05 M NaH_2PO_4, *p*H 8.0, 2 mL). The mixture was stirred for 12 h at room temperature in the dark. After centrifugation at 12000 rpm for 5 min, the isolated solid was washed with PBS for several times, leading to the FITC-Dextran-labeled CALB. For the preparation of Rhodamine B-labelled GOx or HRP, 4 mg mL^{-1} of enzyme (GOx or HRP) solution and 2.5 mg mL^{-1} of Rhodamine B solution were used by a similar method.

Synthesis of mesoporous SBA-15 immobilized system. 5 mg Grubbs catalyst (dissolved in 1.5 mL ethanol) together with 4 mL of CALB solution (0.1 mg mL^{-1}, PBS, pH = 8.0, 50 mM) were mixed with 0.2 g of mesoporous SBA-15. After slowly rotating at 35 °C overnight, the solid material was isolated and the obtained material was dried under vacuum. The obtained material is denoted as Grubbs/CALB@SBA-15.

5. Manipulation of the Interior Architectures

Synthesis of MOF-74 microreactors with different interior capsule size. The dosage of hydrophilic silica emulsifier during the initial emulsification process was varied from 10 to 2.5 and 0.8 wt%. The other procedures were the same as those above.

Synthesis of MOF-74 microreactors with different occupancy of inner compartments. The

volume fractions of the primary O/W emulsion with respect to the confined aqueous phase were varied from 80% to 70%, and 65%, while the other procedures were the same as those above.

Synthesis of MOF-74 microreactors with discrete compartments. The procedures were similar to above, but the primary O/W emulsion was pre-coordinated for 4 h.

6. Preparation of Different Types of MOF Microreactors

For synthesis of Ni-MOF-74-II, MIL-100, MIL-88A, HKUST-1, ZIF-8, and Co-MOF-74@ZIF-67 microreactors, the procedures were similar as above except different metal salts and organic linkers were used. Ni-MOF-74-II, 0.69 mmol $Ni(NO_3)_2{\cdot}6H_2O$, 0.006 and 0.18 mmol 4, 4'-dihydroxybiphenyl-3, 3'-dicarboxylic acid in the inner oil droplets and outer oil continuous phase; MIL-100, 0.37 mmol $FeCl_3{\cdot}6H_2O$, 0.016 and 0.48 mmol 1, 3, 5-benzenetricarboxylic acid in the inner oil droplets and outer oil continuous phase; MIL-88A, 0.37 mmol $FeCl_3{\cdot}6H_2O$, 0.057 and 1.72 mmol fumaric in the inner oil droplets and outer oil continuous phase; HKUST-1, 0.40 mmol $Cu(NO_3)_2{\cdot}3H_2O$, 0.016 and 0.48 mmol 1, 3, 5-benzenetricarboxylic acid in the inner oil droplets and outer oil continuous phase; ZIF-8, 0.336 mmol $Zn(NO_3)_2{\cdot}6H_2O$, 0.081 and 2.44 mmol 2-methylimidazole in the inner oil droplets and outer oil continuous phase; Co-MOF-74@ZIF-67, 0.40 mmol $Co(OAc)_2{\cdot}4H_2O$, 0.012 mmol 2, 5-dihydroxyterephthalic acid and 2.44 mmol 2-methylimidazole in the inner oil droplets and outer oil continuous phase.

7. Encapsulation Ability Measurements

For multi-compartmental MOF system, 5 mg hydrophobic molecular compounds (Grubbs' catalyst or Fe-porphyrin) or 200 μL of protein (CALB, GOx or HRP) stock solution (10 mg mL^{-1}) were pre-dissolved in the inner oil droplets or water compartments. After the formation of MOF skeletons, the leaked proteins in the supernatant were extracted with water. The amount of protein loaded inside multi-compartmental MOF microreactors was determined by UV−Vis spectroscopy by comparing the absorbance of the protein solution before and after immobilization using the Bradford method. The loading efficiency of hydrophobic molecular compounds were determined by ICP−OES. For Pickering double emulsion system, the encapsulation procedures were similar as above, and the amounts of protein or molecular compound were determined by UV−Vis spectroscopy by comparing the absorbance before and after encapsulation. As for the traditional solvothermal system, 200 μL of protein (CALB, GOx or HRP) stock solution (10 mg mL^{-1}) or 5 mg hydrophobic molecular compounds

(Grubbs' catalyst or Fe-porphyrin) were added together with the MOF precursors. After solvothermal treatment at 100 °C, the solid MOF product was isolated and the supernatant was determined by UV−Vis spectroscopy. For the single compartment MOF system, 200 μL of protein (CALB, GOx or HRP) stock solution (10 mg mL^{-1}) was introduced in water phase before the emulsification process, and the leaked enzymes in the supernatant were extracted with water, further determined by UV−Vis spectroscopy by comparing the absorbance of the protein solution before and after encapsulation using standard Bradford method.

8. Permeability Tests

Permeability of enzymes. The MOF microreactors that containing FITC-Dextran-labeled CALB were dispersed into *n*-octanol, and a drop of this solution was placed on the confocal laser scanning microscopy. Then, polar ethanol was slowly added, and the fluorescence variations along with time were recorded.

Permeability of Nile Red molecules. The MOF microreactors were deposited on a glass slide and diluted with octane, followed by addition of 25 μL Nile Red solution (8.0 μM in octane) on the glass slide. The diffusion of Nile Red molecules was recorded by fluorescence microscopy at intervals.

9. Chemo-Enzymatic Cascade Catalysis

Grubb' catalyst/CALB lipase driven ring-closing metathesis/transesterification reaction. Typically, a desired amount of Grubbs/CALB@MOF (200 mg, the loading content of CALB and Grubbs' catalyst were 4 mg g^{-1} and 25 mg g^{-1} respectively) was added into a solution of *n*-hexane (1.0 mL) that containing 0.1 mmol 1,6-heptadien-4-ol and 0.4 mmol vinyl acetate. After fast sealing under N_2 atmosphere, the reaction tube was kept at 45 °C under a stirring rate of 300 rpm. The products were analyzed by GC at intervals. For other catalytic systems, the reaction was conducted under similar conditions except the use of corresponding catalysts. For the recycling experiments, the solid Grubbs/CALB@MOF catalyst (CALB and Grubbs' catalyst loadings are 8 mg g^{-1} and 50 mg g^{-1}) was separated through centrifugation and washed thoroughly with *n*-hexane and dried under vacuum for the next reaction cycle.

GOx/Fe-porphyrin catalyzed oxidation reaction. Specifically, a desired amount of GOx/TPP(Fe)@MOF (200 mg, the loading content of GOx and TPP(Fe) were 7.5 mg g^{-1} and 25 mg

g^{-1} respectively) was added into 5 mL aqueous solution of ABTS (2 mM) and glucose (50 mM), which was then incubated for different reaction time at 30 ºC. Finally, GOx/TPP(Fe)@MOF catalysts were removed from the aforementioned solutions by centrifuge and the resulting supernatants was monitored by UV−vis spectrometry at 415 nm. For other catalytic systems, the reaction was conducted under similar conditions except the use of corresponding catalysts.
